# *Chlamydia*-containing spheres are a novel and predominant form of egress by the pathogen *Chlamydia psittaci*

**DOI:** 10.1128/mbio.01288-24

**Published:** 2024-07-23

**Authors:** Jana Scholz, Gudrun Holland, Michael Laue, Sebastian Banhart, Dagmar Heuer

**Affiliations:** 1 Unit of Sexually Transmitted Bacterial Pathogens and HIV, Robert Koch Institute, Berlin, Germany; 2 Unit of Advanced Light and Electron Microscopy, Robert Koch Institute, Berlin, Germany; Yale University School of Medicine, New Haven, Connecticut, USA; Duke University, Durham, North Carolina, USA

**Keywords:** *Chlamydia psittaci*, *Chlamydia*, egress, obligate intracellular pathogens, cell death, calcium signaling

## Abstract

The egress of intracellular bacteria from host cells and cellular tissues is a critical process during the infection cycle. This process is essential for bacteria to spread inside the host and can influence the outcome of an infection. For the obligate intracellular Gram-negative zoonotic bacterium *Chlamydia psittaci,* little is known about the mechanisms resulting in bacterial egress from the infected epithelium. Here, we describe and characterize *Chlamydia*-containing spheres (CCSs), a novel and predominant type of non-lytic egress utilized by *Chlamydia* spp. CCSs are spherical, low-phase contrast structures surrounded by a phosphatidylserine-exposing membrane with specific barrier functions. They contain infectious progeny and morphologically impaired cellular organelles. CCS formation is a sequential process starting with the proteolytic cleavage of a DEVD tetrapeptide-containing substrate that can be detected inside the chlamydial inclusions, followed by an increase in the intracellular calcium concentration of the infected cell. Subsequently, blebbing of the plasma membrane begins, the inclusion membrane destabilizes, and the proteolytic cleavage of a DEVD-containing substrate increases rapidly within the whole infected cell. Finally, infected, blebbing cells detach and leave the monolayer, thereby forming CCS. This sequence of events is unique for chlamydial CCS formation and fundamentally different from previously described *Chlamydia* egress pathways. Thus, CCS formation represents a major, previously uncharacterized egress pathway for intracellular pathogens that could be linked to *Chlamydia* biology in general and might influence the infection outcome *in vivo*.

**IMPORTANCE:**

Host cell egress is essential for intracellular pathogens to spread within an organism and for host-to-host transmission. Here, we characterize *Chlamydia*-containing sphere (CCS) formation as a novel and predominant non-lytic egress pathway of the intracellular pathogens *Chlamydia psittaci* and *Chlamydia trachomatis*. CCS formation is fundamentally different from extrusion formation, the previously described non-lytic egress pathway of *C. trachomatis*. CCS formation is a unique sequential process, including proteolytic activity, followed by an increase in intracellular calcium concentration, inclusion membrane destabilization, plasma membrane blebbing, and the final detachment of a whole phosphatidylserine-exposing former host cell. Thus, CCS formation represents an important and previously uncharacterized egress pathway for intracellular pathogens that could possibly be linked to *Chlamydia* biology, including host tropism, protection from host cell defense mechanisms, or bacterial pathogenicity.

## INTRODUCTION

*Chlamydia psittaci* is the causative agent of the zoonotic disease psittacosis. Its natural hosts are birds, which can transmit *C. psittaci* to different mammal species, including humans via inhalation of infectious aerosols (1–3). Human infections with *C. psittaci* cause typical pneumonia, with fever, chills, headache, coughing, and dyspnea. However, if left untreated, this infection can lead to severe disease and even death (1–3).

Like other chlamydial species, *C. psittaci* has a biphasic developmental cycle. This cycle is characterized by the presence of infectious, osmotically stable, but mostly metabolically inactive elementary bodies (EBs) and non-infectious, osmotically instable, intracellular replicating reticulate bodies (RBs) (1, 2, 4). Chlamydial infections start with the attachment of an EB to the host cell plasma membrane, where it is endocytosed or phagocytosed by the host cell (4, 5). *C. psittaci* builds its intracellular niche, the inclusion, with the help of specific chlamydial effector proteins. These proteins are secreted via type 3 secretion system (6). At the same time, EBs differentiate into RBs and start to replicate (7). In addition, reorganization of cellular organelles is observed during infection, leading to the fragmentation of the host cell Golgi apparatus and an increase in sphingolipid uptake into the inclusion (8–12). Interfering with ceramide to sphingomyelin (SM) conversion by chemically modified ceramide derivates dramatically decreases the uptake of sphingolipids into the inclusion and infectious progeny formation (13). After bacterial replication and growth of the chlamydial inclusion, RBs redifferentiate into EBs. Finally, the intracellular development of *C. psittaci* is completed by egress from the host cell (5, 14). For different chlamydial species, two forms of egress are described: a lytic egress and a non-lytic egress pathway called extrusion formation (14, 15). Extrusion formation has been predominantly studied for the human pathogen *Chlamydia trachomatis*, but only rarely for *C. psittaci*. In *C. trachomatis*, extrusion formation depends on a cytoskeleton- and calcium-dependent release process of the membrane-enclosed bacteria without host cell death (15–17). In addition, RBs can spread between cells using tunneling nanotubes (18).

The egress of intracellular pathogens is of general importance for the host because it usually induces the inflammatory response of the host. In comparison to other life cycle phases of intracellular pathogens, host cell egress is still understudied, and thus, the identification and characterization of novel egress strategies continue (19, 20). In principle, egress is defined as the release of progeny to infect a new host cell. Flieger et al. (20) distinguish between three principal egress mechanisms: (i) active host cell destruction, (ii) induced membrane-dependent egress without host cell destruction, and (iii) induced programmed host cell death (20). This induced programmed host cell death can be apoptosis, pyroptosis, or necroptosis (20). During egress by active host cell destruction, the intracellular pathogens are released into the extracellular space. In addition, membrane- or cell death-dependent egress mechanisms exist, where pathogens are released into a membranous compartment to spread between cells without contact with the extracellular environment. This could protect the pathogen from extracellular immune defense mechanisms (19, 20). Apoptosis as an egress mechanism has been reported for the Gram-positive bacterium *Mycobacterium marinum*, the Gram-negative bacterium *Salmonella enterica,* the protozoan parasite *Leishmania* spp., and the fungal pathogen *Cryptococcus neoformans* (20–22). Interestingly, 25 years ago, it was reported that *C. psittaci* infection causes the induction of apoptosis in late infections (23). However, a link between *Chlamydia*-induced apoptosis and *C. psittaci* egress was not investigated. In general, the findings about the role of different cell death pathways during chlamydial infections are still controversial, and the role of apoptosis during chlamydial infections is still under investigation (24–30).

In this article, we present a new egress mechanism of *C. psittaci* that involves the formation of *Chlamydia*-containing spheres (CCSs). This pathway is observed after detecting proteolytic activity mainly in late *C. psittaci* inclusions and an increase in cytosolic calcium concentration in infected cells. Following the increase in calcium, the plasma membrane begins to bleb, and the inclusion membrane destabilizes. CCSs are formed by the detachment of infected cells. The resulting CCSs contain cellular organelles, including *Chlamydia*, and expose phosphatidylserine at the surrounding membrane. Our results suggest that *Chlamydia* spp. exit host cells by three mutually exclusive pathways: host cell lysis, extrusion, and the newly described CCS.

## MATERIALS AND METHODS

### Cell culture, transient transfection, and infection assays

Cell culture, transient transfection, and infection assays were performed as described previously (12). For further information, see Text S1.

### Live cell imaging of *C. psittaci* and *C. trachomatis* egress

To monitor *C. psittaci* or *C. trachomatis* egress, live cell imaging of *Chlamydia*-infected cells stably expressing eGFP was performed. For more details, see Text S1.

### Separation of CCS and free bacteria for reinfection assay

To separate CCS and free bacteria, HeLa cells were initially infected with *C. psittaci* and incubated for 48 h. Then, supernatants were collected and centrifuged at low speed (5 min, 300 × *g*, RT) to pellet CCS and separate them from free bacteria in the remaining supernatant. Subsequently, CCS and free bacteria were subjected to glass bead lysis followed by infection of freshly seeded HeLa cells. Cells were fixed at 24 h post-infection (pi), stained for Hsp60 (see Immunofluorescence assay for more details), and the numbers of infection-forming units (IFU) per mL were calculated from average inclusion counts in 10 fields of view per condition.

### Quantitative real-time PCR

Determination of bacterial genome copy numbers by quantitative real-time PCR (qRT-PCR) was performed as already described (31) using samples from the reinfection assay.

### Isolation and staining of CCS

CCSs in the supernatant of *C. psittaci*-infected cell cultures were separated by centrifugation (5 min, 300 × *g*, RT) at 48 h pi. For live staining, the pellet was mixed with the indicated staining solutions. The preparation of the staining solutions and the staining procedures are described in Text S1. For immunofluorescence staining, the pellet was mixed with 4% paraformaldehyde in phosphate-buffered saline (PBS) and transferred into a poly-l-lysine coated 8-well-chambered coverslip (µ-Slide 8 Well, Ibidi). After 30 min of incubation at RT, staining was continued as described for immunofluorescence assays.

### Induction of apoptosis by treatment with staurosporine

Apoptosis was induced by treatment with 10 µM staurosporine (Biomol). At 42 h post-treatment, apoptotic cells in the supernatant were collected by centrifugation (5 min, 300 × *g*, RT). Staining of apoptotic cells and preparation for transmission electron microscopy (TEM) were performed as described for CCS.

### Immunofluorescence assay

For immunofluorescence assays, HeLa or A549 cells were seeded onto cover slips and infected with *C. psittaci*, *C. trachomatis* (MOI 2), or left uninfected. At indicated time points, the culture medium was removed, and cells were fixed with 4% paraformaldehyde in PBS (30 min, RT). After washing with PBS, samples were blocked and permeabilized using 0.2% Triton X‐100 and 0.2% BSA in PBS (20 min, RT). Incubation with primary antibodies (1 h, RT) was followed by washing and incubation with secondary antibodies and DAPI (25 µg mL^−1^, Sigma-Aldrich) (1 h, RT). All antibodies were diluted using 0.2% BSA in PBS. The used antibody concentrations are listed in Text S1. All samples were analyzed with a Stellaris 8 Confocal Microscope (Leica Microsystems).

### Transmission electron microscopy

Adherent HeLa cells, which were infected with *C. psittaci* (MOI 2) for 48 h, the culture supernatant of *C. psittaci*-infected cell cultures (sedimented by centrifugation for 5 min at 300 × *g*), and adherent uninfected HeLa cells (42 h after apoptosis induction) were fixed with a mixture of glutaraldehyde and formaldehyde (2.5% and 1%, respectively, in 50 mM HEPES buffer). After incubation for 2 h at RT, cells were scraped from the dishes and collected in HEPES buffer. All suspensions were centrifuged (6,000 × *g*, 10 min), and sediments were embedded in low-melting-point agarose. Small sample blocks were extracted from the solidified agarose, post-fixed in osmium tetroxide (1% in distilled water), followed by block contrasting with tannic acid (0.1% in 50 mM HEPES buffer) and uranyl acetate (2% in distilled water). Subsequently, samples were dehydrated by incubation in a graded ethanol series and embedded in epon resin. After polymerization for 48 h at 60°C, ultrathin sections (approximately 70 nm) were prepared using an ultramicrotome (UC7; Leica) and stained with uranyl acetate (2% in distilled water, 20 min), followed by lead citrate (2 min) to increase contrast. Sections were examined with a transmission electron microscope (Tecnai 12, FEI Corp.) at 120 kV. Images were recorded using a CMOS camera (Phurona, emsis). Overview images of entire section profiles of CCS, infected HeLa cells, or apoptotic cells were acquired by using the image montaging mode of the camera software (Radius, emsis). Images were aligned and stitched by using Microsoft Image Composite Editor. Contrast and brightness were adjusted using Adobe Photoshop. For quantification, TEM images of thin sections through fixed and embedded supernatants of *C. psittaci*-infected HeLa cells were analyzed for the different *Chlamydia*-containing cellular structures present (see Fig. S2 for an overview of the analyzed structures). In total, 582 structures were analyzed in three independent biological replicates.

### Quantification of CCS or extrusions

The number of CCS or extrusions in *C. psittaci-* or *C. trachomatis*-infected cell cultures, respectively, was determined under different inhibitory and medium conditions. Information about the different inhibitory and medium conditions, experimental procedures, and determination of the working concentrations of the caspase inhibitors are given in Text S1.

### Imaging of cytosolic sphingomyelin exposure

To image the cytosolic SM exposure at the inclusion membrane, we used a HeLa cell line stably expressing a non-toxic, HaloTagged version of equinatoxin II (EqtSM-HaloTag) as cytosolic SM reporter (32). For more details, see Text S1.

### Determination of proteolytic DEVD cleaving activity

Cells were cultured and infected with *C. psittaci* (MOI 2) in 8-well-chambered coverslips (µ-Slide 8 Well, Ibidi). Proteolytic DEVD-cleaving activity was determined using the Incucyte Caspase-3/7 Red Dye for Apoptosis (Sartorius) as described in the manufacturer’s protocol. For more details, see Text S1.

### Calcium imaging

Imaging of intracellular calcium levels and distribution was performed by either Rhod 3 staining or live cell imaging of the dual calcium reporter cell line HeLa ER-LAR-Geco G-Geco (33). Both methods are described in more detail in Text S1.

## RESULTS

### *C. psittaci* egresses from the cellular monolayer through the formation of *Chlamydia*-containing spheres

**Fig 1:**
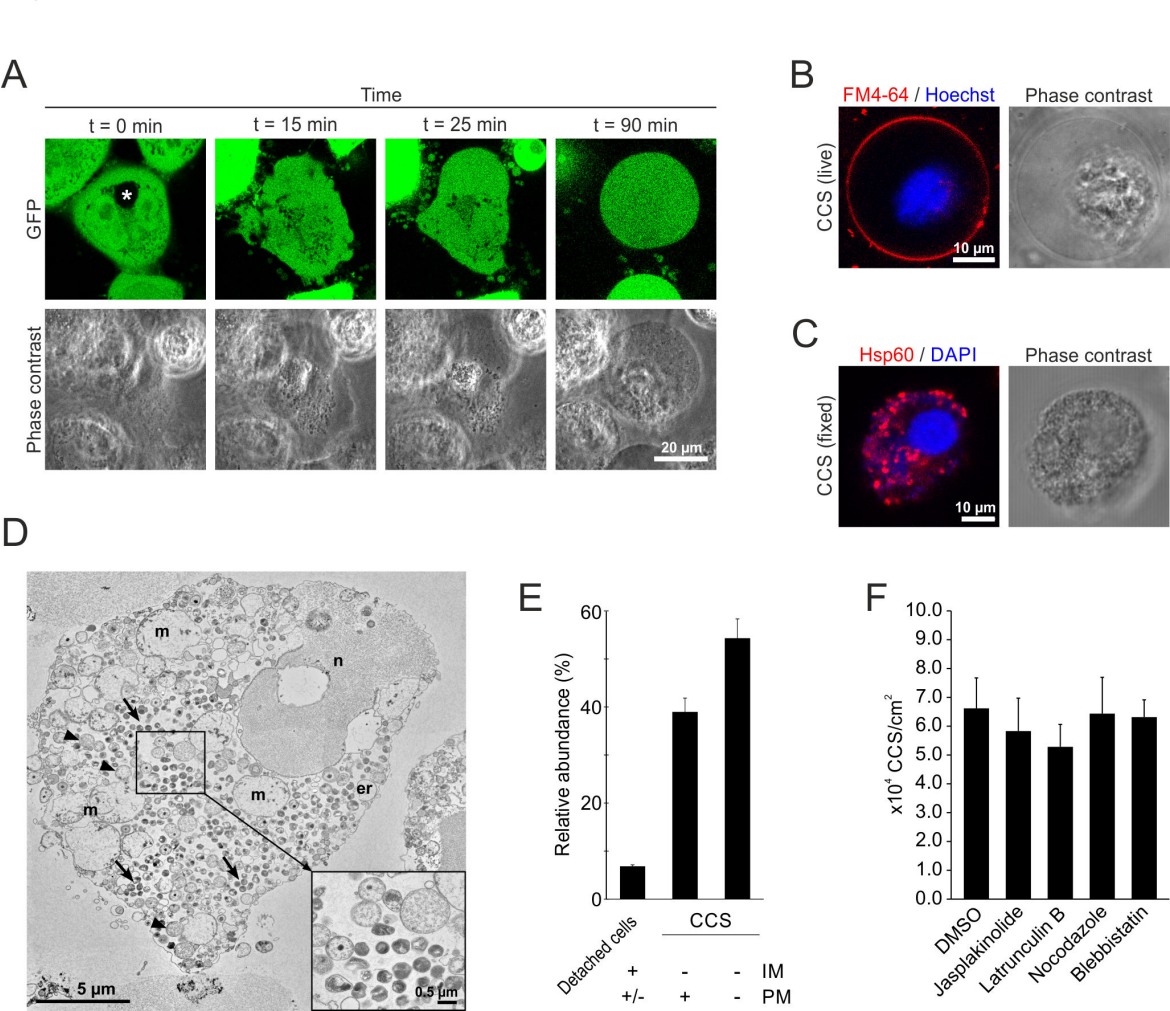
CCS formation represents the predominate *C. psittaci* egress pathway and is characterized by membrane blebbing and destabilization of the inclusion membrane. (**A**) Time course of CCS formation. *C. psittaci*-infected HeLa cells (MOI 2) stably expressing GFP were monitored starting at 44 h pi using a confocal laser scanning microscopy equipped with a live-cell chamber. Panels show representative images of a section plane of a CCS-forming HeLa cell at 45 h pi; *n* = 2. Asterisk indicates *C. psittaci* inclusion. (**B**) Representative fluorescence images of a live CCS isolated from the supernatant of *C. psittaci*-infected HeLa cells (MOI 2, 48 h pi). The surrounding membrane was visualized using the membrane marker FM 4-64, and DNA was counterstained by Hoechst; *n* = 3. (**C**) Representative fluorescence images of a paraformaldehyde-fixed CCS isolated from *C. psittaci*-infected HeLa cells (MOI 2, 48 h pi). Bacteria inside the CCS were detected using a chlamydial Hsp60 (Cy3) antibody, and the DNA was counterstained using DAPI; *n* > 3. (D) Transmission electron microscopy of a thin section through a chemically fixed CCS isolated from the supernatant of *C. psittaci*-infected HeLa cells (MOI 2, 48 h pi). The CCS is almost entirely surrounded by an intact membrane and reveals many RBs (arrowheads) and EBs (arrows) besides structurally impaired cell organelles, such as mitochondria (M) or the nucleus (N). The inset shows a small region from the center of the CCS at higher magnification with bacteria at different stages of differentiation; *n* = 3 experiments. (**E**) Quantification of the CCSs and detached cells found in the supernatant of *C. psittaci*-infected HeLa cell cultures. TEM images of thin sections through fixed and embedded supernatants of *C. psittaci*-infected HeLa cells (MOI 2, 48 h pi) were analyzed for the different structures present (see Fig. S2 for an overview of some of the structures). IM, inclusion membrane and PM, plasma membrane. Data show mean ± SEM of three independent experiments. (**F**) CCS formation is not influenced by inhibitors of host cell cytoskeleton elements. HeLa cells were infected with *C. psittaci* at MOI 2, washed with PBS at 44 h pi, and treated with 1 µM jasplakinolide, 0.5 µM latrunculin B, 30 µM nocodazole, and 50 µM blebbistatin or DMSO (negative control), and stained with Hoechst. *C. psittaci* CCS in the supernatant were quantified at 48 h pi. Data show mean ± SEM; *n* ≥ 3; *P < 0.05 (Student’s *t-*test).

To investigate *C. psittaci* egress in greater detail, we monitored HeLa cells that stably expressed eGFP in the cytosol after infection with *C. psittaci* using fluorescence live cell microscopy, starting at the end of the chlamydial developmental cycle (45 h pi). Initially, the bacterial inclusion did not contain any eGFP as indicated by a black hole in the eGFP-detecting channel (Fig. 1A). Then, blebbing of the cellular plasma membrane was observed, followed by the influx of eGFP into the inclusion lumen, demonstrating loss of inclusion membrane integrity. Subsequently, the plasma membrane blebs enlarged, and the entire host cell detached, forming spherical, low-phase-contrast structures in the supernatant of the infected cell cultures. We named these structures *Chlamydia*-containing spheres (Fig. 1A; Movie S1).

To differentiate between the release of *C. psittaci* through CCS and extrusion formation of *C. trachomatis*, we conducted additional fluorescence live-cell microscopic analysis using HeLa cells stably expressing cytosolic eGFP that were infected with *C. trachomatis* L2. Unlike CCS formation, the release of *C. trachomatis* L2 through extrusions resulted in the inclusion remaining eGFP negative throughout the process, supporting previous observations that the inclusion membrane remains intact in extrusions (15). Additionally, during extrusion formation, the host cell remained intact and did not detach (Movie S2).

These results indicate that the exit of *C. psittaci* from the host cell occurs through the formation of CCSs, which are clearly distinguishable from *C. trachomatis* extrusions both morphologically and in their formation.

To further describe the morphology of *C. psittaci* CCS, we stained CCS with different markers and analyzed stained CCS by confocal laser scanning microscopy (CLSM). CCSs were stained with FM 4-64, a dye that stains the plasma membrane and then integrates into the cellular vesicular network, and Hoechst. Fluorescence live cell confocal microscopy of stained CCSs showed that CCSs were surrounded by a membrane and contained concentrated DNA, suggesting the presence of the cell nucleus (Fig. 1B). CLSM was used to examine immunofluorescence staining of fixed CCS. Antibodies specific for bacterial Hsp60 were used, along with the DNA marker DAPI, showing that bacteria positive for both markers were dispersed throughout the CCS, while concentrated, Hsp60-negative DNA next to the bacteria supports the presence of a host cell nucleus within CCS (Fig. 1C). In contrast, extrusions formed by *C. trachomatis* L2 expressing eGFP and stained with Hoechst were filled with bacteria that were positive for both GFP and Hoechst. No other host cell structures were positive for Hoechst (Fig. S1).

We next characterized CCS by thin section TEM to resolve CCS morphology at the ultrastructural level (Fig. 1D). TEM showed that CCSs are bounded by a more or less intact plasma membrane and contain *C. psittaci* EBs and fewer RBs, which are dispersed throughout the CCS. The bacteria intermingle with morphologically impaired cell organelles, such as mitochondria, endoplasmic reticulum, and nucleus, and are not separated from them by an inclusion membrane (Fig. 1D). Quantification of *C. psittaci*-containing cellular structures found in the supernatant of *C. psittaci*-infected cell cultures showed that 93% of the structures were CCS, while 7% were detached cells (Fig. 1E). In the detached cells, bacteria were surrounded by an inclusion membrane, and 84% of the cells showed signs of cell death (apoptosis or necrosis) with or without preserved plasma membrane. No inclusion membrane was found in any of the CCS detected, and the CCS showed no signs of apoptosis. About 39% of the CCSs were surrounded by a plasma membrane, while 54% revealed only fragments of the plasma membrane around the CCS, suggesting that the stability of the plasma membrane was affected by CCS formation (Fig. 1E; Fig. S2). Besides the structures described above, the supernatant showed detached cells without any bacteria and aggregates of cellular debris with and without bacteria (Fig. S2). The data presented indicate that CCSs are distinct from extrusions in terms of their structural and ultra-structural characteristics. The formation of *C. trachomatis* extrusions depends on different parts of the host cell cytoskeleton (15). To investigate the requirement of the host cell cytoskeleton for CCS formation, we treated *C. psittaci*-infected cell cultures between 44 and 48 h pi with the previously described inhibitors jasplakinolide, latrunculin B, nocodazole, and blebbistatin as inhibitors of actin depolymerization, actin polymerization, microtubules, neural Wiskott-Aldrich syndrome protein, and myosin II, respectively. We observed no significant difference in the number of CCS formed between DMSO-treated controls (6.6 × 10^4^ CCS/cm^2^) and jasplakinolide-, latrunculin B-, nocodazole-, and blebbistatin-treated samples (5.8, 5.3, 6.4, and 6.3 × 10^4^ CCS/cm^2^, respectively) (Fig. 1F). This was in contrast to late *C. trachomatis* infections where we observed an increase in extrusion formation for jasplakinolide- and nocodazole-treated samples (0.78 and 0.51 × 10^4^ extrusions/cm^2^, respectively) compared to DMSO-treated controls (0.49 × 10^4^ extrusions/cm^2^) and a decrease in extrusion formation for latrunculin B- and blebbistatin-treated samples (0.27 and 0.00 × 10^4^ extrusions/cm^2^, respectively) (Fig. S3A), consistent with the findings of Hybiske and Stephens (15).

In humans, *C. psittaci* predominantly infects the respiratory tract (1, 2). To investigate whether CCSs are also formed after infection of lung tissue cell lines or not, we studied *C. psittaci* infections in the lung tissue cell line A549. At first, we showed that A549 cells can be infected with *C. psittaci* by detecting inclusions using immunofluorescence staining for the chlamydial antigens Hsp60 and *C. psittaci-*specific IncA. *C. psittaci* inclusions filled with Hsp60-stained *Chlamydia* and surrounded by an IncA-stained inclusion membrane were detected at 24 and 48 h pi (Fig. S3B). Microscopic analysis of supernatants of infected A549 cells at 48 h pi revealed spherical structures with a diameter of 25–30 µm, which we identified as CCS (Fig. S3C).

Collectively, these data demonstrate that *C. psittaci* egresses from cell culture monolayers by CCS formation, a novel non-lytic egress pathway that differs morphologically and mechanistically from other non-lytic bacterial exit pathways including extrusions.

### CCS formation is the predominant egress pathway of *C. psittaci* and is also present, but less frequent, for *C. trachomatis*

**Fig 2:**
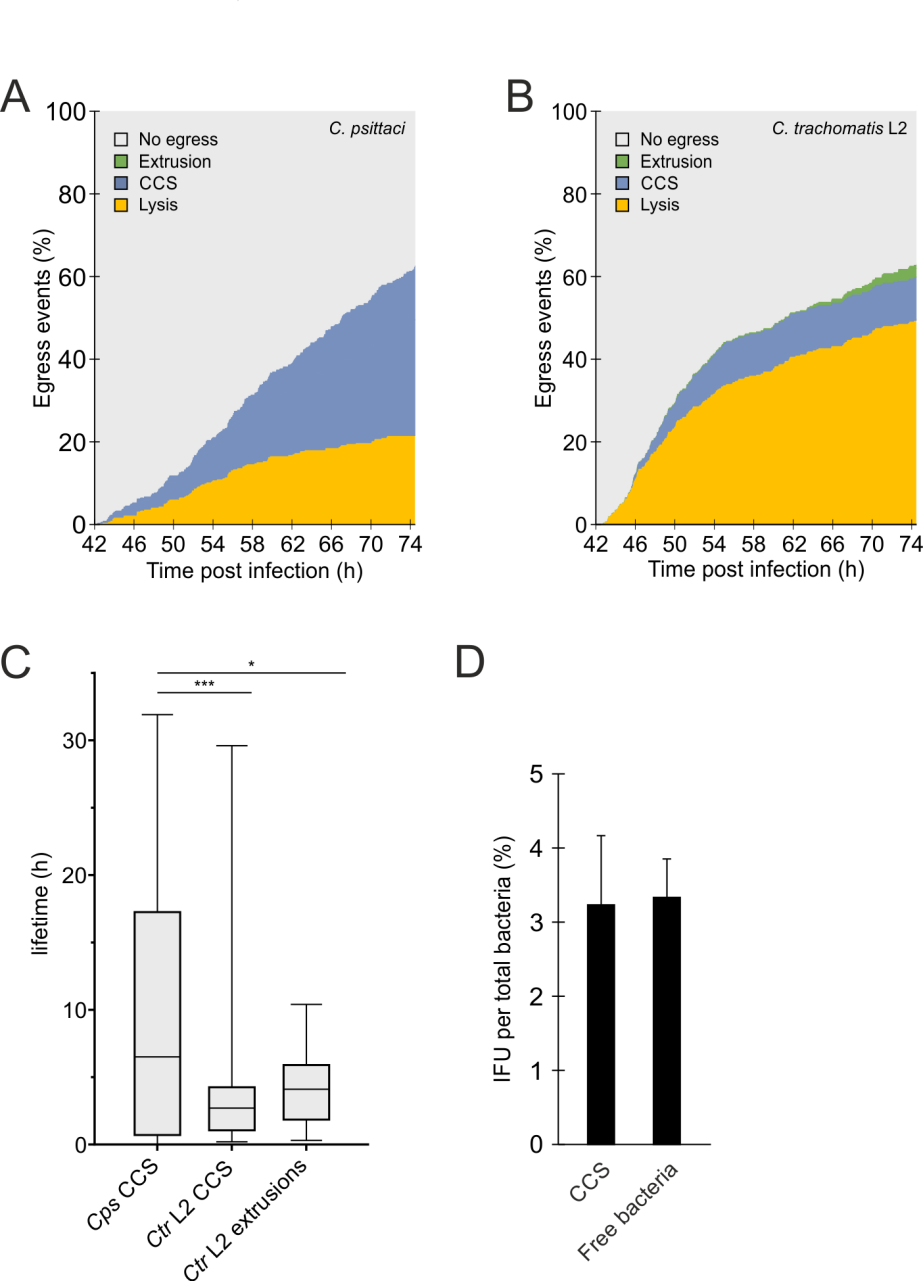
CCS formation is the predominant egress pathway of *C. psittaci* and is also present, but less frequent, for *C. trachomatis*. (**A**) Frequency and time dependence of different *C. psittaci* egress pathways. HeLa cells stably expressing GFP were infected with *C. psittaci* (MOI 2), and at 42 h pi, Z-stack images were acquired using a CLSM equipped with a live-cell chamber for 32.5 h. The type of egress pathway (CCS formation, lysis, extrusion formation, or no egress) and time point of a total of 410 cells (*n* = 2 biological replicates) were analyzed. Data show the part of each egress pathway type on the total amount of infected cells at each given time point. (**B**) Frequency and time dependence of different *C. trachomatis* L2 egress pathways. HeLa cells stably expressing GFP were infected with *C. trachomatis* L2 (MOI 2), and at 42 h pi, Z-stack images were acquired using a CLSM equipped with a live-cell chamber for 32.5 h. The type of egress pathway (CCS formation, lysis, extrusion formation, or no egress) and time point of 391 cells (*n* = 2 biological replicates) were analyzed. Data show the part of each egress pathway type on the total amount of infected cells at each given time point. (**C**) Stability of *C. psittaci* and *C. trachomatis* L2 CCS and extrusions. Lifetime of all observed *C. psittaci* and *C. trachomatis* L2 CCS and extrusions (see Fig. 2A and B) was determined. Data show the median lifetime as a box and whisker plot. *n* = 3; *P < 0.05 (Student’s *t*-test). (**D**) CCSs preserve the infectivity of *C. psittaci*. CCSs in the supernatant of *C. psittaci*-infected HeLa cells (MOI 2) were separated from free bacteria by centrifugation (300 × *g*, 5 min, RT) at 48 h pi. Infectious progeny was titrated after glass bead lysis, and numbers were normalized to genome copy numbers determined by qRT-PCR. Data show mean ± SEM; *n* = 3.

During *C. trachomatis* infection of HeLa cell culture monolayers, host cell lysis and extrusion formation occur at nearly identical frequencies at 72 h pi (15). To compare this later time point of infection with *C. psittaci* infections, we monitored HeLa cells infected with *C. psittaci* that stably expressed eGFP in the cytosol by live cell microscopy from 42 to 74.5 h pi. The frequencies of CCS formation, extrusion formation, and host cell lysis were quantified at each time point pi (Fig. 2A). Throughout the observation period, we observed a nearly linear increase of egress events over time with 63% of bacteria egressed at the end of the observation period. CCS formation and host cell lysis were constantly observed, while extrusion formation was not observed in *C. psittaci* infections. At the end of the observation period, 41.2% of the infected cells formed CCS and 21.5% underwent host cell lysis (for a representative movie of a *C. psittaci*-infected cell undergoing host cell lysis, see Movie S3).

To investigate whether CCS formation is specific to *C. psittaci* or also occurs during *C. trachomatis* infections, we used live cell microscopy of *C. trachomatis* L2-infected, eGFP-expressing HeLa cells from 42 to 74.5 h pi. Interestingly, we observed *C. trachomatis*-infected cells forming a CCS at the end of the developmental cycle (Movie S4). However, CCS formation was less frequent for *C. trachomatis* compared to *C. psittaci*, with only 10.5% of the observed infected cells formed a CCS. In addition, CCS formation of *C. trachomatis*-infected cells mainly occurs between 45 and 55 h pi (Fig. 2B). By 74.5 h post-infection, 63% of the *C. trachomatis* had egressed. Of the infected cells, 10.5% formed CCS, 49.4% underwent host cell lysis (for a representative movie of a *C. trachomatis* infected cell undergoing host cell lysis, see Movie S5), and 3.1% formed an extrusion. At late infection time points, between 70.5 and 74.5 h pi, we observed that 42% of *C. trachomatis* exited the host cell through extrusion formation, while 58% of infected cells lysed. At this time point, we did not detect any CCS formation, which supports previous findings by Hybiske and Stephens (15) (Fig. S4).

Live cell imaging was used to track the stability of both CCS and extrusions in eGFP-expressing HeLa cells following their formation. For *C. psittaci*, approximately one-third of the formed CCSs underwent lysis at the end of the observation period, while two-thirds remained stable (data not shown). The median time to lysis for *C. psittaci* CCSs was approximately 6.5 h with an interquartile range (IQR) of 0.7–17.3 h (Fig. 2C). For *C. trachomatis* L2 extrusions, approximately one-third of the formed extrusions underwent lysis at the end of the observation period, with a median lifetime of approximately 4.1 h (IQR: 1.8–5.5 h) (Fig. 2C). In contrast, CCS from *C. trachomatis* L2-infected cells underwent lysis more frequently, with approximately 99.7% of them being lysed at the end of the observation period. The stability of *C. trachomatis* L2 CCS was lower than that of *C. psittaci*, with a median time to lysis of 2.7 h (IQR: 1.0–4.1 h) (Fig. 2C).

To determine if CCSs contain infectious bacteria capable of infecting new host cells, we collected the supernatant from infected cells and isolated CCS from free bacteria released by host cell or CCS lysis using differential centrifugation. We then quantified the infectious progeny after glass bead lysis and normalized the IFU counts to bacterial genome copy numbers. The percentage of infectious progeny in CCS was 3.2% ± 0.9%, which was comparable to that of free bacteria (3.3% ± 0.5%) (Fig. 2D).

In summary, these data show that CCS formation is the predominant non-lytic egress pathway of *C. psittaci*. Additionally, CCS formation is also present but less frequent for *C. trachomatis* L2. The CCSs in the supernatant contain infectious bacteria and are stable for several hours, indicating that CCSs are relevant non-lytic egress structures of *C. psittaci* and *C. trachomatis*.

### The exposure of sphingolipids in the *C. psittaci* inclusion membrane to the cytosol precedes its destabilization and subsequent CCS formation

One important feature of the formation of extrusions in *C. trachomatis* L2 is the consistent stability of the inclusion membrane, which contrasts with the destabilization of the inclusion membrane during host cell lysis (15). Notably, we observed the influx of eGFP into the inclusion lumen during CCS formation (Fig. 1A). Therefore, we further examined changes in the inclusion membrane during CCS formation. To achieve this, we utilized an engineered, non-toxic variant of equinatoxin II (EqtSM), a recently established reporter for SM. EqtSM has previously been used to investigate membrane damage induced by pathogens and the vacuolar escape of *Mycobacterium marinum* and *Salmonella enterica* (32, 34, 35). The recruitment of EqtSM to the pathogen-containing vacuole is linked to SM rearrangements in the vacuolar membrane, which is then followed by its lysis.

**Fig 3:**
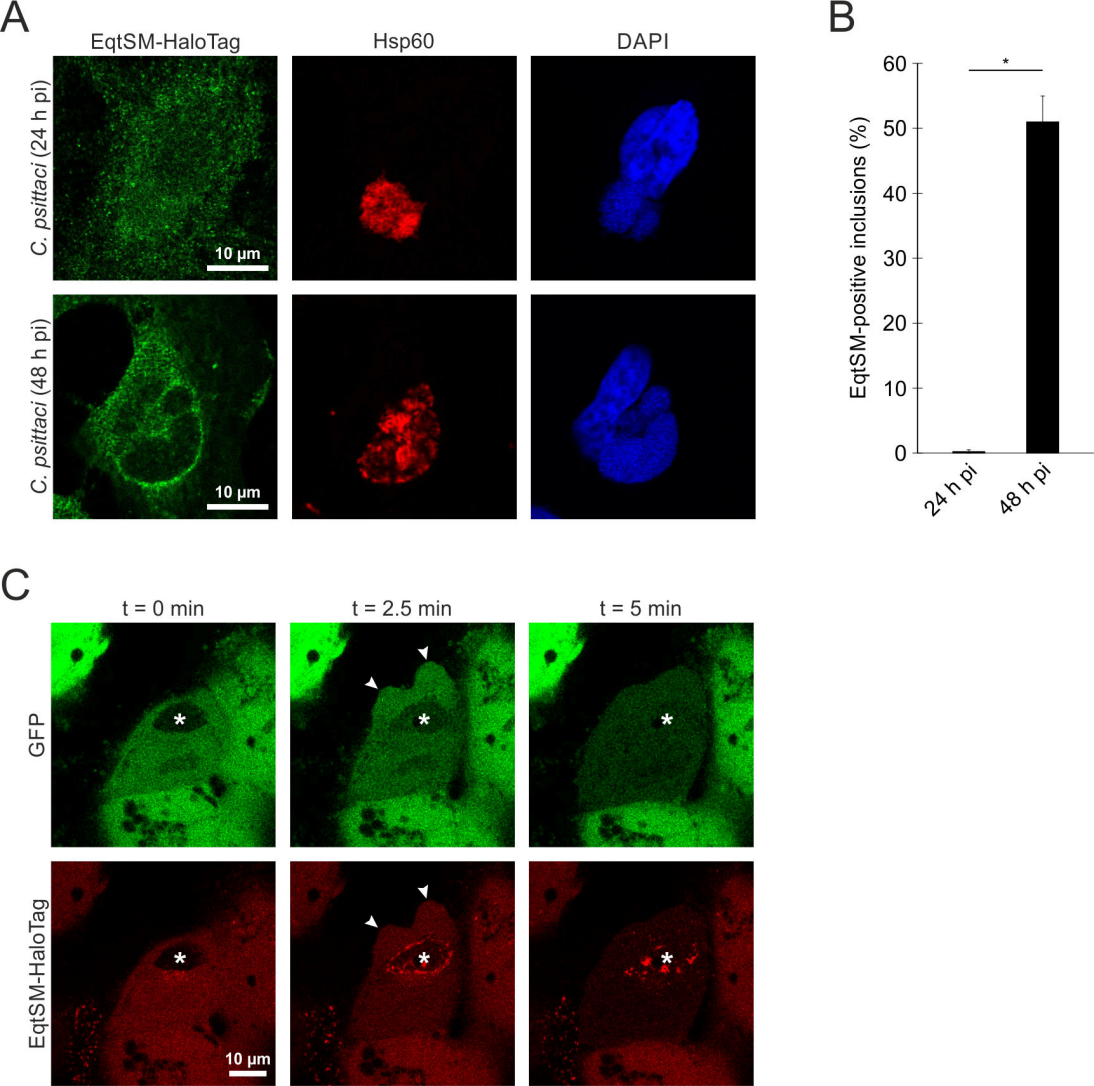
During CCS formation, sphingolipids in the *C. psittaci* inclusion membrane are reorganized, followed by its destabilization and CCS formation. (**A**) Recruitment of EqtSM-HaloTag to the *C. psittaci* inclusion occurs at 48 h pi. Representative fluorescent images of *C. psittaci*-infected HeLa cells stably expressing EqtSM-HaloTag at 24 and 48 h pi. HeLa cells stably expressing EqtSM-HaloTag were infected with *C. psittaci* (MOI 2) and fixed at 24 or 48 h pi with PFA. *C. psittaci* and EqtSM-HaloTag were detected using a mouse-anti-Hsp60 (Cy3) and a rabbit-anti-HaloTag (AF488) antibody, respectively. The DNA was counterstained using DAPI; *n* = 4. (**B**) EqtSM-HaloTag is recruited to approximately 51% of the *C. psittaci* inclusions at 48 h pi, but hardly any at 24 h pi. Immunofluorescent images of *C. psittaci*-infected HeLa cells stably expressing EqtSM-HaloTag (MOI 2) at 24 or 48 h pi were analyzed for the recruitment of EqtSM-HaloTag to the *C. psittaci* inclusion. Data show mean ± SEM; *n* = 4; *P < 0.05 (Student’s *t*-test). (**C**) EqtSM recruitment is followed by permeabilization of the inclusion membrane and CCS formation. HeLa cells stably expressing EqtSM-HaloTag were transiently transfected with a plasmid for cytosolic expression of eGFP and infected with *C. psittaci* (MOI 2). At 44 h pi, the cells were labeled with 200 nM Janelia Fluor 585 HaloTag Ligand (Promega) and monitored using a CLSM equipped with a live-cell chamber. Panels show representative images of an EqtSM-HaloTag recruiting *C. psittaci* inclusion during CCS formation, which subsequently gets permeable for eGFP; *n* = 3. Asterisks mark *C. psittaci* inclusion, and arrowheads indicate blebbing of the host cell plasma membrane.

Therefore, we analyzed the recruitment of stably expressed HaloTagged EqtSM (EqtSM-HaloTag) in HeLa cells to mid- and late*-C. psittaci* inclusions at 24 and 48 h pi, respectively, as an indicator of inclusion membrane instability. Using immunofluorescence staining with a HaloTag antibody, we observed the recruitment of EqtSM-HaloTag to approximately 51.0% of *C. psittaci* inclusions at 48 h pi, but hardly any at 24 h pi (Fig. 3A and B).

We investigated whether EqtSM-HaloTag recruitment correlates in time with inclusion membrane destabilization and CCS formation. To do this, we infected HeLa cells that stably express EqtSM-HaloTag and transiently express eGFP with *C. psittaci*. At 44 h pi, we labeled the cells with Janelia Fluor 585 HaloTag Ligand and performed live cell imaging. We observed recruitment of EqtSM-HaloTag just before host cell plasma membrane blebbing (arrowheads) was detected and eGFP influx into the inclusion lumen occurred (Fig. 3C).

Taken together, these data indicate that sphingolipids in the inclusion membrane of *C. psittaci* undergo reorganization, and this reorganization correlates in time with inclusion membrane destabilization and the onset of CCS formation.

### CCSs differ from apoptotic cells by retaining their membrane integrity and by lacking fragmentation of the nucleus

We aimed to better characterize the molecular mechanisms leading to CCS formation. About 20 years ago, it was shown that an atypical form of apoptosis gets activated during late *C. psittaci* infection, but the function of this form of apoptosis for *C. psittaci* infection remains unclear (23). By live cell microscopy, we detected plasma membrane blebbing during CCS formation, which is described as a characteristic feature of apoptosis (36, 37). Thus, we asked whether CCS formation is linked to the activation of apoptosis. During early apoptosis, phosphatidylserine is exposed to the outer leaflet of the plasma membrane (38, 39). In addition, at late stages of apoptosis, secondary necrosis can be induced, leading to the loss of plasma membrane integrity (40, 41).

**Fig 4:**
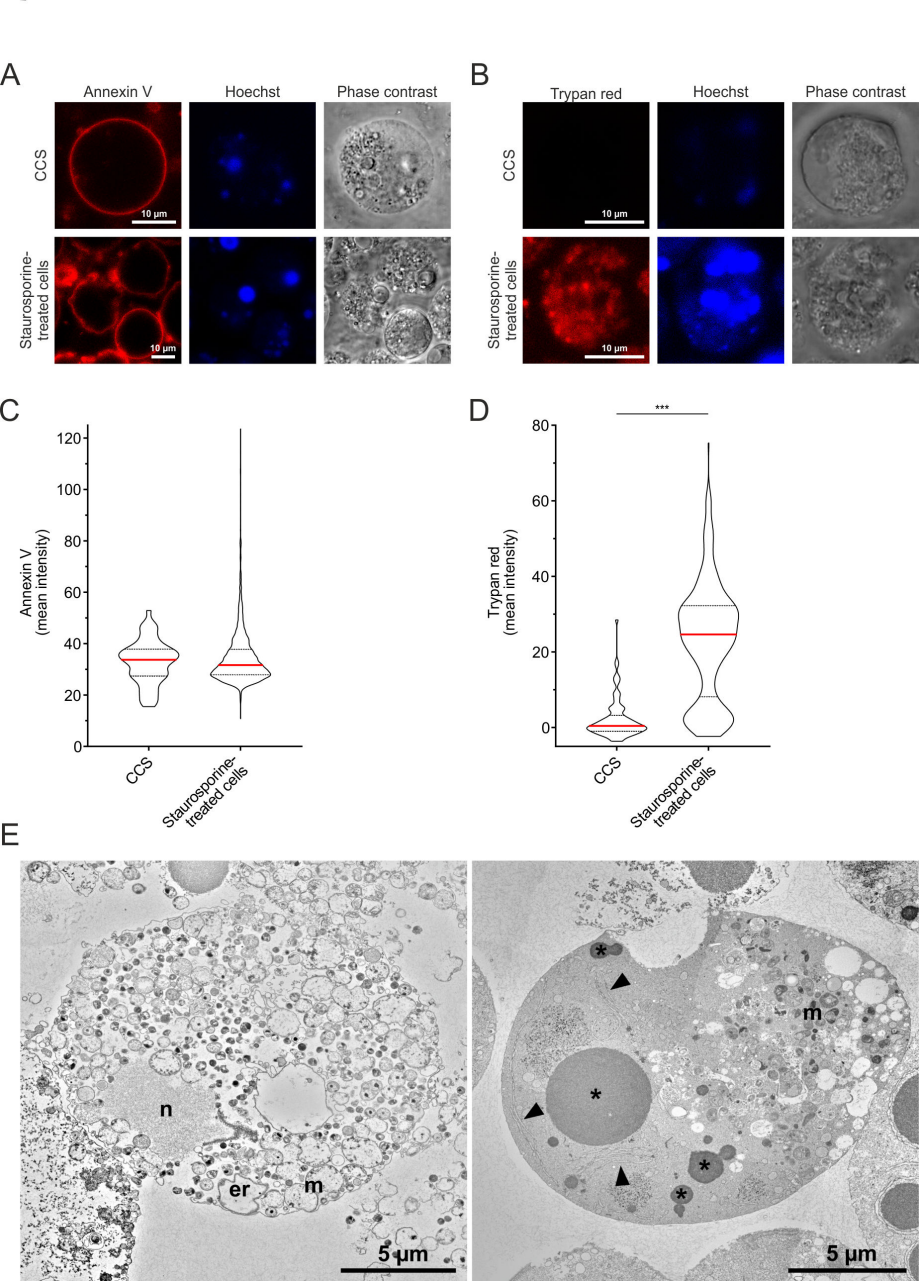
CCSs retain membrane integrity, and the nucleus and mitochondria are not condensed in contrast to apoptotic cells. (**A and B**) Representative images of a CCS positive for Annexin V (Alexa Fluor 568) (**A**), but not for TrypanRed Plus (**B**) in comparison to staurosporine-treated, apoptotic cells positive for both. CCSs were collected from the supernatant of *C. psittaci*-infected HeLa cells (MOI 2, 48 h pi), while apoptotic cells were collected from the supernatant of uninfected, staurosporine-treated HeLa cells (10 µM staurosporine, 42 h post-treatment). PS was visualized using Annexin V (Alexa Fluor 568), structures with damaged or compromised membranes were stained with TrypanRed Plus, and DNA was counterstained using Hoechst. (**C**) Quantification of PS exposure of CCS and staurosporine-treated, apoptotic cells. Mean intensities of the Annexin V staining of individual Hoechst-positive CCSs and apoptotic cell structures of at least 2 µm^2^ were determined, and the distribution of the mean intensities of 54 CCS and 2,213 apoptotic cell structures was visualized as violin plot; *n* = 3. (**D**) Quantification of membrane integrity of CCS and staurosporine-treated, apoptotic cells. Mean intensities of the TrypanRed Plus staining of individual CCSs and single apoptotic cells were determined, and the distribution of the mean intensities of 83 CCS and 404 apoptotic cells was visualized as violin plot; *n* = 3; ***P < 0.005 (Student’s *t*-test). (**E**) Transmission electron microscopy of thin sections through a CCS isolated from the supernatant of *C. psittaci*-infected HeLa cells (MOI 2, 48 h pi) (left image) and a staurosporine-treated, apoptotic cell (right image). The apoptotic cell reveals a dense cytoplasm with one larger and a few smaller profiles of the fragmented nucleus (asterisk). The matrix of the nuclear fragments appears dense and homogenous and nuclear membranes are detached (arrowheads; see also Fig. S6). The CCS shows many bacterial profiles at different stages (RBs, EBs, and intermediate forms) and cellular organelles, such as nucleus (N), mitochondria (M), and the endoplasmic reticulum (er), which appear extracted and dilated (see Fig. S6C and D for details).

To investigate if CCSs show any of these apoptosis characteristics, we stained CCS and staurosporine-treated apoptotic cells as control with Annexin V as phosphatidylserine marker and TrypanRed Plus as a marker of plasma membrane integrity. CCS showed a clear membrane staining with Annexin V (Fig. 4A); however, no TrypanRed Plus staining was observed within CCS (Fig. 4B), which was also confirmed by SYTOX Green staining (Fig. S5A). In contrast, staurosporine-treated apoptotic cells were positive for all indicated markers (Fig. 4A and B; Fig. S5A).

Next, we investigated the distribution of the marker intensities between the populations of CCS and apoptotic cells. For this, we visualized the mean intensities of the Annexin V staining of individual CCS and apoptotic cell structures as violin plot. Both populations were distributed around a median of 33.7 and 31.7 for CCS and staurosporine-treated cells, respectively, with similar quartiles of 27.5 and 37.6 for CCS and 27.8 and 37.7 for apoptotic cells (Fig. 4C). Additionally, we visualized the mean intensities of the TrypanRed Plus staining of individual CCSs and apoptotic cells as violin plot. Here, the median for CCS of 0.4 was significantly lower than the median for apoptotic cells of 24.6 with quartiles of −0.9 and 3.2 for CCS and 8.3 and 32.1 for apoptotic cells (Fig. 4D). Similar to TrypanRed Plus staining, quantification of the SYTOX Green intensities showed that the median for CCS of 0.2 was significantly lower than the median for apoptotic cells of 6.7 with quartiles of −0.1 and 1.4 for CCS and 0.0 and 14.1 for apoptotic cells (Fig. S5B).

We also compared the ultrastructure of CCS- and staurosporine-treated apoptotic cells by thin-section TEM. While apoptotic cells showed a dense cytoplasm with fragmented nuclei and aberrant mitochondria, CCSs contained lysed and dilated cell organelles, such as mitochondria, endoplasmic reticulum, and the nucleus (Fig. 4E; Fig. S6).

Taken together, these results indicate that the CCS are morphologically different from apoptotic cells.

### CCS formation correlates in time with a proteolytic activity specific for a DEVD-containing substrate independent of caspase activation

**Fig 5:**
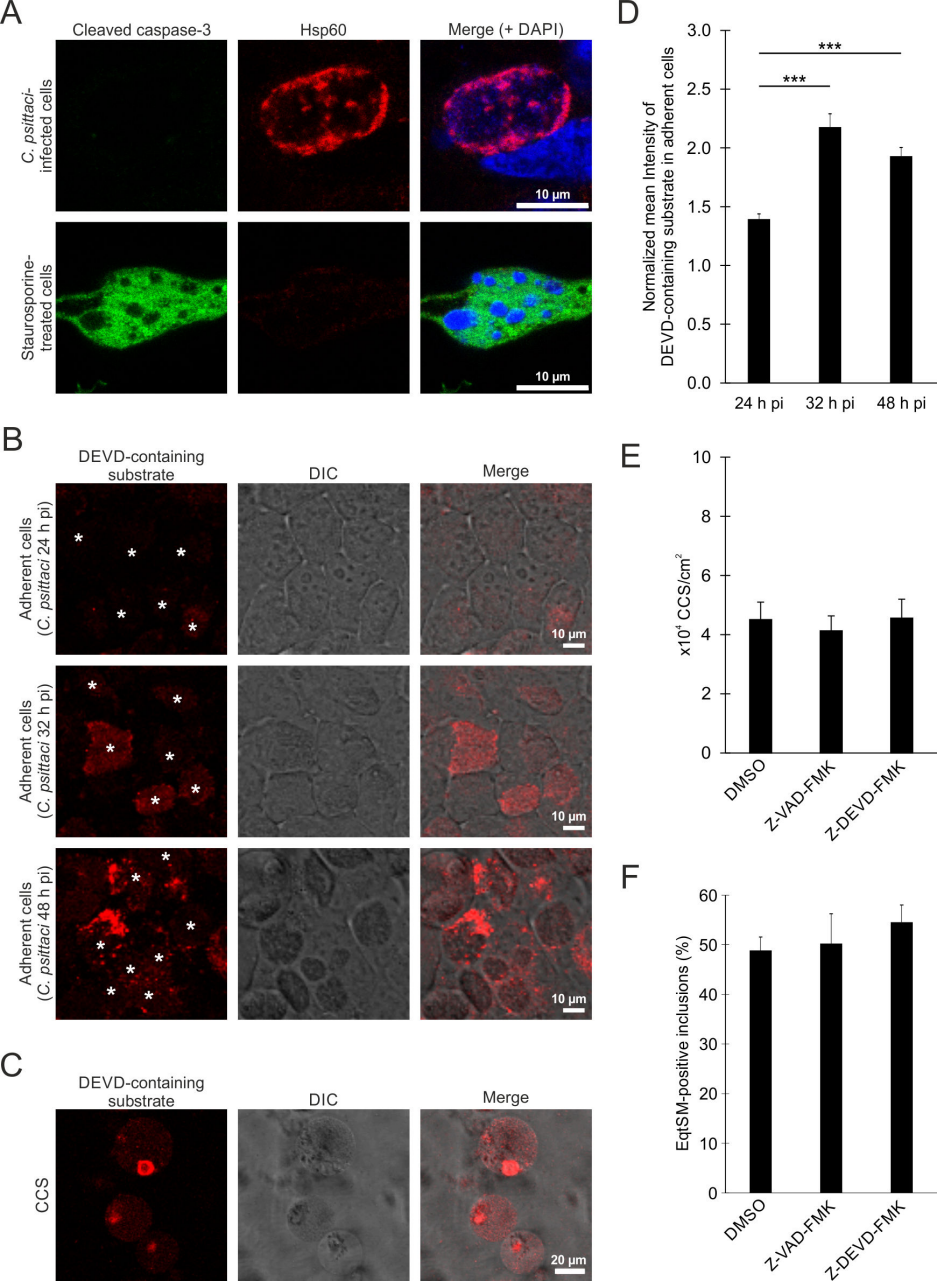
CCS formation is independent of caspases, while a DEVD-containing substrate is proteolytically cleaved during CCS formation. (**A**) No cleavage of caspase-3 is detectable at late *C. psittaci* infections. Representative fluorescence images of *C. psittaci*-infected HeLa cells (MOI 2, 48 h pi) and staurosporine-treated uninfected HeLa cells as positive control. Cells were fixed with PFA, and *C. psittaci* and cleaved caspase-3 were detected using a mouse-anti-Hsp60 (Cy3) and a rabbit-anti-cleaved caspase-3 (AF488) antibody, respectively. The DNA was counterstained using DAPI; *n* = 2. (**B and C**) *C. psittaci* infection leads to the activation of the caspase detection reagent by proteolytic cleavage of a DEVD-containing substrate. HeLa cells were infected with *C. psittaci* (MOI 2), and at 40 h pi, the cells were stained with Incucyte Caspase-3/7 Dye for Apoptosis. Representative images of the infected, adherent cells at 24, 32, and 48 h pi (**B**) and CCS in the supernatant at 48 h pi (**C**) are shown; *n* = 3. (**D**) Quantification of the proteolytic activity during CCS formation. *C. psittaci*-infected HeLa cells (MOI 2) were stained with Incucyte Caspase-3/7 Dye for Apoptosis at 16, 24, or 40 h pi for 8 h. Z-stack images were acquired at a CLSM. Data were normalized to mean fluorescence intensity of uninfected cells at respective time points. Data show mean ± SEM; *n* = 3; ***P < 0.005 (Student’s *t*-test). (**E**) CCS formation is not affected by caspase inhibition. *C. psittaci-*infected HeLa cells (MOI 2) were washed with PBS at 44 h pi and treated with 2 µM Z-VAD-FMK, 2 µM Z-DEVD-FMK, or DMSO (negative control). CCSs in the supernatant were quantified at 48 h pi. Data show mean ± SEM; *n* = 3. (**F**) EqtSM-HaloTag recruitment is not affected by caspase inhibition. *C. psittaci-*infected HeLa cells stably expressing EqtSM-HaloTag (MOI 2) were washed with PBS at 44 h pi and treated with 2 µM Z-VAD-FMK, 2 µM Z-DEVD-FMK, or DMSO (negative control), and cells were fixed at 48 h pi with PFA. *C. psittaci* and EqtSM-HaloTag were detected using a mouse-anti-Hsp60 (Cy3) and a rabbit-anti-HaloTag (AF488) antibody, respectively. The DNA was counterstained using DAPI. *C. psittaci* inclusions were analyzed for the recruitment of EqtSM-HaloTag. Data show mean ± SEM; *n* = 3.

The intrinsic and extrinsic pathways of apoptosis converge in caspase-3 activation (36). Therefore, we analyzed the presence of cleaved caspase-3 in *C. psittaci*-infected cells at 48 h post-infection by immunofluorescence staining using an antibody specific for activated caspase-3. However, we did not detect activated caspase-3 in the cytosol of *C. psittaci*-infected host cells at 48 h post-infection, whereas uninfected, staurosporine-treated cells showed clear staining of activated caspase-3 (Fig. 5A). Interestingly, we observed cleavage of the DEVD-containing substrate at 32 h pi mainly in *C. psittaci* inclusions using a fluorescent caspase-3/7 detection reagent that detects proteolytic cleavage of the substrate. The substrate was less present at 24 h pi (Fig. 5B). At 48 h pi, the cleavage of the DEVD-containing substrate was mainly observed in the cytosol of infected cells (Fig. 5B) and in CCS (Fig. 5C), indicating the involvement of a DEVD-containing substrate cleaving protease in CCS formation. We also quantified the proteolytic cleavage of DEVD in adherent cells. Toward the end of the chlamydial developmental cycle, we observed a significant increase in intensity. At 24 h pi, the intensity was 1.4-fold higher compared to uninfected cells. At 32 and 48 h pi, the intensity was 2.2- and 1.9-fold higher, respectively, compared to uninfected cells (Fig. 5D).

We investigated whether inhibiting caspase-3 affects CCS formation. To do this, we used Z-VAD-FMK as a pan-caspase inhibitor and Z-DEVD-FMK as a specific caspase-3 inhibitor. Both inhibitors were used at a concentration of 2 µM to prevent caspase 3 activation during staurosporine-induced apoptosis (Fig. S7). After treatment with Z-VAD-FMK and Z-DEVD-FMK for 44–48 h post-infection, there was no significant difference in the number of CCS between the DMSO-treated controls (4.5 × 10^4^ CCS/cm^2^) and the Z-VAD-FMK- and Z-DEVD-FMK-treated samples (4.1 and 4.6 × 10^4^ CCS/cm^2^, respectively) (Fig. 5E). Additionally, we analyzed EqtSM-HaloTag recruitment after treatment with Z-VAD-FMK and Z-DEVD-FMK for 44–48 h post-infection. We did not observe a significant difference in the proportion of EqtSM-HaloTag recruiting *C. psittaci* inclusions between DMSO-treated controls (48.9% of inclusions) and Z-VAD-FMK- and Z-DEVD-FMK-treated samples (50.2% and 54.5% of inclusions, respectively) (Fig. 5F). Furthermore, after treatment with Z-DEVD-FMK, proteolytic cleavage of DEVD was still observed in infected cells (Fig. S8). These data indicate that a DEVD-containing substrate is cleaved within *C. psittaci* inclusions prior to CCS formation, but CCS formation is independent of classical apoptosis.

### The concentration of calcium in the cytosol increases before the formation of CCS

Calcium is an important signaling molecule involved in diverse cellular processes (42–44). Furthermore, calcium has been described to regulate the egress of *C. trachomatis* (15, 16). Therefore, we investigated the role of calcium in the formation of CCS.

**Fig 6:**
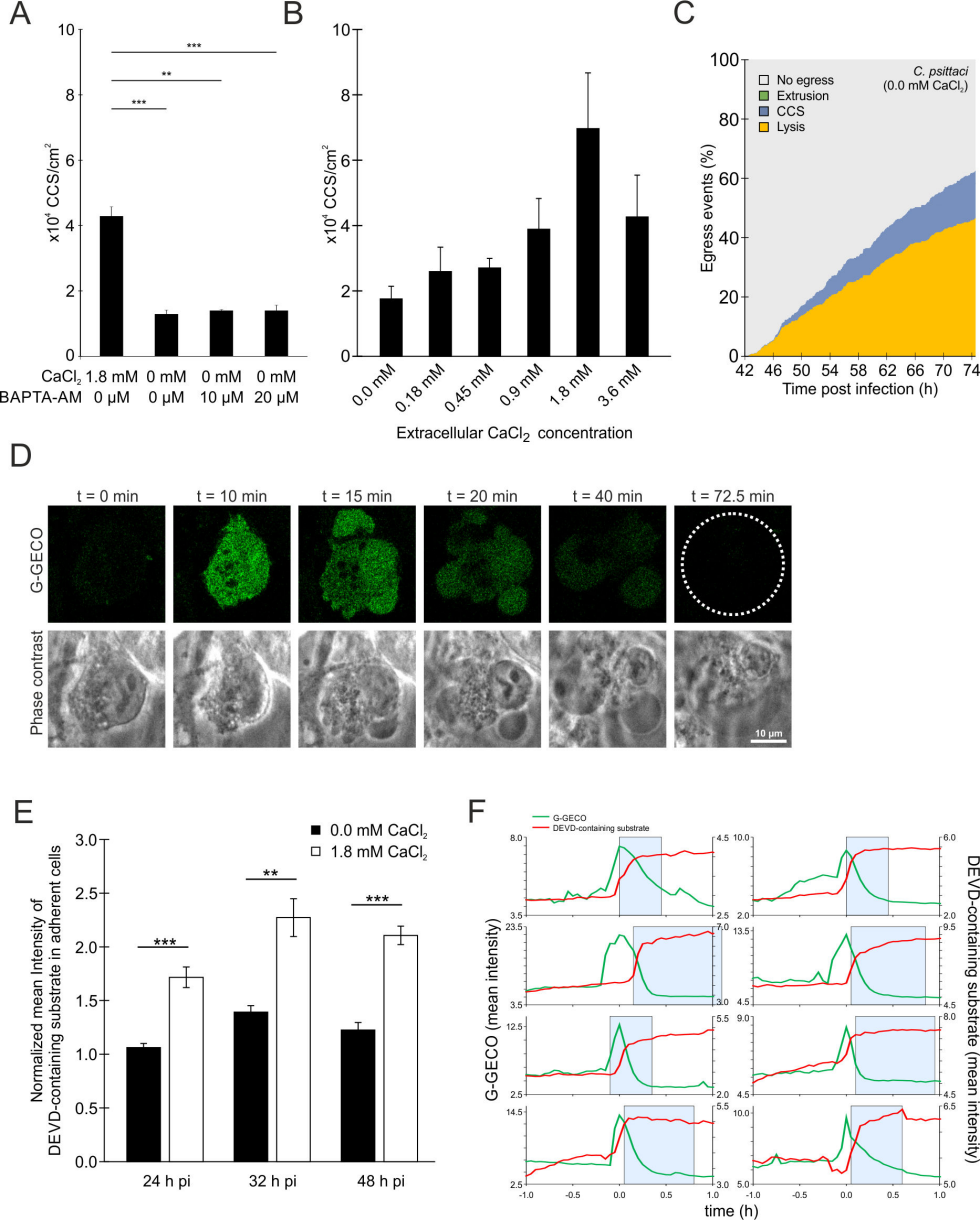
CCS formation is calcium dependent. (**A**) CCS formation is decreased under calcium chloride-depleting conditions in the culture medium, but not further decreased by the addition of the cellular calcium chelator BAPTA-AM. HeLa cells were infected with *C. psittaci* (MOI 2), and at 44 h pi, the culture medium was replaced with serum-free medium supplemented with the indicated concentrations of calcium chloride and BAPTA-AM. CCSs in the supernatant were visually quantified at 48 h pi. Data show mean ± SEM; *n* = 3; **P < 0.01 and ***P < 0.005 (Student’s *t*-test). (**B**) CCS formation depends on the extracellular calcium concentration. HeLa cells were infected with *C. psittaci* (MOI 2). At 44 h pi, the culture medium was replaced with serum-free medium supplemented with the indicated concentrations of calcium chloride. CCSs in the supernatant were visually quantified at 48 h pi. Data show mean ± SEM; *n* = 4. (**C**) Frequency and time dependence of different *C. psittaci* egress pathways under calcium depletion. HeLa cells stably expressing GFP were infected with *C. psittaci* (MOI 2), and at 42 h pi, the culture medium was replaced with serum-free and calcium chloride-free medium, and Z-stack images were acquired using a CLSM equipped with a live-cell chamber for 32.5 h. The type of egress pathway (CCS formation, lysis, extrusion formation, or no egress) and time point of a total of 427 cells (*n* = 2 biological replicates) were analyzed. Data show the part of each egress pathway type on the total amount of infected cells at each given time point. (**D**) Intracellular calcium concentration increases during CCS formation. Dual calcium reporter HeLa ER-LAR-Geco G-Geco cells were infected with *C. psittaci* (MOI 2). At 44 h pi, cells were monitored at a CLSM equipped with a live-cell chamber. Panels show representative images of a CCS-forming cell. Dashed line indicates CCS; *n* = 3. (**E**) Proteolytic DEVD-cleaving activity in *C. psittaci*-infected HeLa cells depends on the extracellular calcium concentration. Culture medium of *C. psittaci*-infected HeLa cells (MOI 2) was replaced with serum-free medium supplemented with indicated concentrations of calcium chloride at 20, 28, and 44 h pi, and cells were stained with Incucyte Caspase-3/7 Dye for Apoptosis for 4 h. Z-stack images were acquired using a CLSM. Data were normalized to mean fluorescence intensity of uninfected cells without calcium chloride at respective time points. Data show mean ± SEM; *n* = 3; **P < 0.01 and ***P < 0.005 (Student’s *t*-test). (**F**) During CCS formation, proteolytic DEVD-cleaving activity rapidly increases after increase of the intracellular calcium concentration. Dual calcium reporter HeLa ER-LAR-Geco G-Geco cells were infected with *C. psittaci* (MOI 2). At 44 h pi, culture medium was replaced with serum-free medium supplemented with 1.8 mM calcium chloride, and cells were stained with Incucyte Caspase-3/7 Dye for Apoptosis. Cells were monitored at a CLSM equipped with a live-cell chamber for 4 h. Data show the cytosolic calcium concentration and proteolytic DEVD-cleaving activity of eight individual CCS-forming cells of two biological replicates. The start and end of membrane blebbing are indicated by a blue box.

Initially, we investigated the impact of reducing extracellular and intracellular calcium levels on CCS formation. We cultured cells infected with *C. psittaci* in a medium containing calcium chloride or in a calcium chloride-free medium. We then introduced the cell-permeable calcium chelator BAPTA-AM at varying concentrations between 44 and 48 h pi. Reducing the extracellular calcium concentration from 1.8 mM (a concentration identical to our standard infection medium) to 0.0 mM decreased CCS formation from 4.3 to 1.3 × 10^4^ CCS/cm^2^, respectively (Fig. 6A). Additional use of BAPTA-AM in concentrations of 10 and 20 µM to the calcium chloride-free medium did not further decrease CCS formation (1.4 and 1.4 × 10^4^ CCS/cm^2^, respectively). In addition, we cultivated *C. psittaci*-infected cells with calcium chloride concentrations ranging from 0 to 1.8 mM and from 44 to 48 h pi. We observed a dose-dependent increase in CCS formation until 1.8 mM calcium chloride (from 1.8 to 7.0 × 10^4^ CCS/cm^2^), while CCS numbers decreased at 3.6 mM calcium chloride to 4.3 × 10^4^ CCS/cm^2^ (Fig. 6B).

We next asked whether the decrease in the extracellular calcium concentration shifts egress toward host cell lysis or prevents egress in general. To address this question, we monitored *C. psittaci*-infected HeLa cells stably expressing eGFP in the cytosol in calcium chloride-free medium by live cell microscopy from 42 to 74.5 h pi and determined frequencies of the different egress pathways (Fig. 6C). Compared to calcium chloride-containing conditions (Fig. 1B), the frequency of CCS formation decreased from 41.2% to 15.9%, while the frequency of host cell lysis increased from 21.5% to 46.6%. The frequency of *C. psittaci*-infected cells showing no sign of egress was similar at 37.5% compared to 37.3% for calcium-containing conditions, indicating that *C. psittaci*-infected cells underwent host cell lysis instead of CCS formation under calcium chloride-free conditions.

To better understand the impact of varying extracellular calcium concentrations on intracellular calcium levels, we examined the intracellular calcium concentration during the advanced stages of *C. psittaci* infection. We used the Rhod-3 membrane-permeant cytosolic calcium sensor under conditions containing 0.0 and 1.8 mM calcium chloride. At 0.0 mM calcium chloride, we did not detect an increase in cytosolic calcium concentration in *C. psittaci*-infected cells at 48 h pi. However, at 1.8 mM calcium chloride, we observed *C. psittaci*-infected cells with blebbing membranes and an increased cytosolic calcium concentration (Fig. S9). Therefore, we aimed to determine the chronological order of membrane blebbing and calcium influx. We visualized intracellular calcium levels during CCS formation using a genetically encoded calcium sensor (33). In HeLa cells that stably express G-Geco, an increase in intracellular calcium concentration was detected during CCS formation before the initiation of the CCS characteristic membrane blebbing. This increased calcium concentration is subsequently slowly reduced during CCS formation, leading to a CCS with basal calcium levels again (Fig. 6D).

Next, we addressed whether extracellular calcium concentration affected the cleavage of the DEVD-containing substrate late in *C. psittaci*-infected cells. For this, we compared the DEVD-cleaving activity at 1.8 mM calcium chloride-containing conditions to calcium-free conditions using the caspase-3/7 detection reagent. At all examined time points (24, 32, and 48 h pi), we observed a decrease in the intensity from 1.7- to 1.1-fold, 2.3- to 1.4-fold, and 2.1- to 1.2-fold, respectively (Fig. 6E). We aimed to further link the observed increase in cytosolic calcium concentration with the events of CCS formation, membrane blebbing, and cleavage of the DEVD-containing substrate. For this, we measured the DEVD-cleaving activity using the caspase-3/7 detection reagent in G-Geco cells simultaneously with the cytosolic calcium concentration at 1.8 mM calcium chloride-containing medium conditions. Analysis of individual CCS-forming cells revealed that after a rapid increase in the cytosolic calcium concentration, DEVD-cleaving activity also rapidly increased (Fig. 6F), showing that an increase in cytosolic calcium concentration precedes the activation of a DEVD-containing protease. Interestingly, while the increase in cytosolic calcium concentration was rather transient, DEVD-cleaving activity remained stable for a longer time period.

In summary, our data support that calcium is an important regulator in *Chlamydia* biology, particularly in controlling the egress pathways of *C. psittaci* and *C. trachomatis*.

## DISCUSSION

The release of infectious bacteria from an infected host cell or tissue is crucial for intracellular bacteria to complete their infection cycle and spread within or transmit to a new host. Variations in egress pathways have been associated with specific aspects of pathogen biology, such as transmission, host tropism, and pathogenicity (20, 27, 45, 46). Therefore, understanding the egress strategies employed by different pathogens is as important as comprehending the adhesion and invasion processes. This understanding provides deeper insights into the biology of a specific pathogen. It is interesting to note that little is known about the egress mechanisms of the zoonotic bacterial pathogen *C. psittaci*.

**Fig 7:**
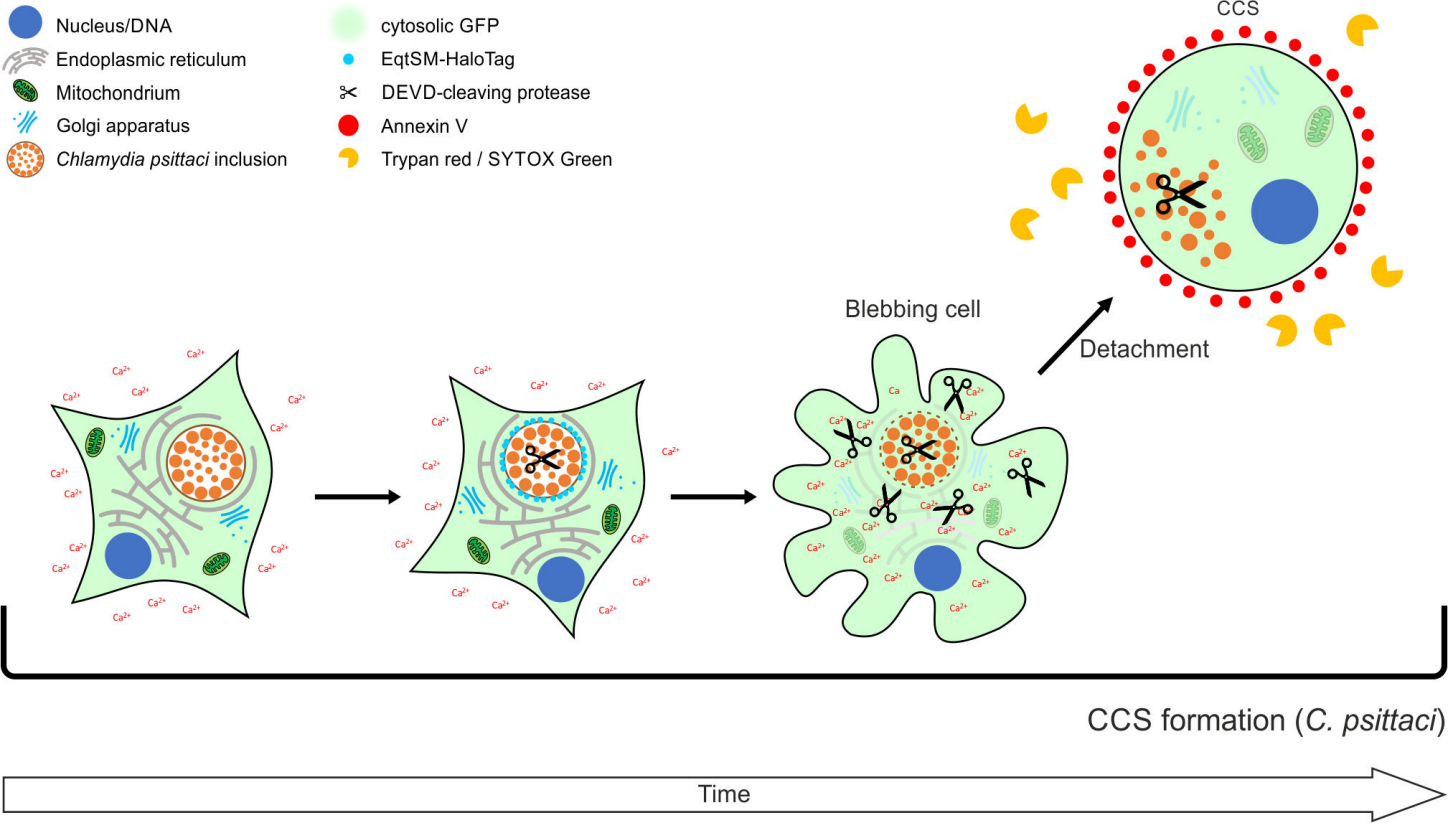
Graphical model of *C. psittaci* CCS formation. CCS formation begins after an increase in the cytosolic calcium concentration, sphingolipid rearrangement, and destabilization of the inclusion membrane, plasma membrane blebbing, and increased activation of a DEVD-cleaving protease. The whole blebbing cell detaches and forms a spherical, *Chlamydia*-containing structure, which we termed *Chlamydia*-containing sphere. CCSs contain dispersed *Chlamydia* in EB and RB stages, morphologically impaired cell organelles, and are surrounded by a membrane. This membrane presents PS to its extracellular surface but retains its membrane barrier function.

In this study, we demonstrate that *C. psittaci* predominantly uses host cell-derived *Chlamydia*-containing spheres as non-lytic egress structures to exit epithelial cell cultures. CCSs are present in the supernatant of infected cell monolayers and are enclosed by a single membrane derived from the host cell plasma membrane. The surrounding membrane retains selective membrane barrier functions and exposes phosphatidylserine on the outer leaflet. In addition to infectious EBs and RBs, CCSs also contain host cell DNA and morphologically impaired host cell organelles, including the nucleus. For members of the family *Chlamydiaceae*, egress has mainly been studied in the strict human pathogen *C. trachomatis*. It has been shown that *C. trachomatis* can egress the infected epithelial host cell by lysis of the host cell or by extrusion formation, a non-lytic egress pathway. Although CCS and extrusions are both non-lytic egress pathways, they are distinct in terms of morphology and formation. CCSs are formed by a sequence of events (Fig. 7). First, proteolytic cleavage of a DEVD-containing substrate can be detected inside the chlamydial inclusions, followed by an increase in the intracellular calcium concentration of the infected cell. Subsequently, blebbing of the plasma membrane begins, the inclusion membrane exposes SM to the cytosol and then destabilizes, and the proteolytic cleavage of the DEVD-containing substrate increases rapidly within the whole infected cell. Finally, infected, blebbing cells detach and leave the monolayer resulting in CCS in the supernatant.

In contrast to CCS, *C. trachomatis* extrusion formation is characterized as a packaged release process without host cell death, where the intact *C. trachomatis*-filled inclusion is packed into a protrusion depending on the host cell cytoskeleton. This protrusion is then pinched off from the surviving host cell by a mechanism depending on the host cell cytoskeleton, but not depending on proteases (15–17). Formed extrusions of *C. trachomatis* are covered by two membrane layers consisting of both the intact inclusion membrane and the plasma membrane of the host cell separated by a thin cytoplasmic layer (15, 47). In addition, only 15.3% of *C. trachomatis* extrusions show extensive surface exposure of phosphatidylserine, while 59.6% of extrusions show a punctate phosphatidylserine pattern and 24.4% of extrusions do not expose phosphatidylserine (47). Furthermore, *C. trachomatis* extrusions are free of host cell nuclei and rarely consist of other host cellular organelles, which is substantial for the survival of the remaining host cell (15, 47). It is noteworthy that the formation of CCS was observed not only during *C. psittaci* infections but also during *C. trachomatis* infections, albeit with a lower frequency. However, these *C. trachomatis* CCSs were formed only until 55 h pi and were less stable compared to *C. psittaci* CCS. In contrast, *C. trachomatis* extrusion formation became more prominent after 65 h pi and showed similar frequencies to host cell lysis during the last hours of our observation. These results are in good agreement with previously published data describing similar frequencies of *C. trachomatis* extrusion formation and lysis at 72 h pi. Furthermore, our data extend our knowledge about *C. trachomatis* egress via CCS formation at earlier infection time points.

It is noteworthy that a clear distinction exists between CCS and extrusions, with the former exhibiting the preservation of the inclusion and inclusion membrane. The inclusion represents a protective intracellular niche that supports chlamydial growth. Therefore, the lysis of the inclusion membrane must be regulated during the chlamydial cycle of development to avoid premature inclusion membrane lysis, which would activate host cell death pathways and limit the formation of infectious EB. A defining feature of CCS formation is the destabilization of the inclusion membrane, as evidenced by the influx of eGFP to the inclusion lumen. This step in CCS formation was further characterized using cytosolic EqtSM, an engineered, non-toxic version of equinatoxin II, as an SM reporter (34). Similar to pathogen-induced membrane damage and vacuolar escape of *Mycobacterium marinum* and *Salmonella enterica*, SM in the inclusion membrane is exposed to the cytosol during CCS formation of *C. psittaci* prior to inclusion membrane destabilization (32, 35). This suggests that a rearrangement of the sphingolipids in the inclusion membrane results in its destabilization, thereby indicating that sphingolipids may influence CCS formation. Interfering with the expression of specific *C. trachomatis* Inc-proteins by generating *C. trachomatis* mutants induces premature lysis of the inclusion membrane (26, 48–50). Recent studies by Cortina and Derré (51) showed that the ER-localized calcium sensor STIM1 plays a role in stabilizing the *C. trachomatis* inclusion membrane (51, 52). They demonstrate that the *C. trachomatis* inclusion membrane protein IncS_Ct_ (CTL0402) recruits STIM1 to the *C. trachomatis* inclusion membrane, while the *C. muridarum* analog IncS_Cm_ failed to recruit STIM1 (51, 53). Furthermore, *C. trachomatis* strains lacking IncS_Ct_ perform higher rates of inclusion lysis at late developmental stages, supporting previous findings using *C. trachomatis* and *C. muridarum* chimeras (49). In addition, Nguyen et al. (16) showed that depletion of STIM1 reduced extrusion formation in *C. trachomatis*, indicating that stabilization of the inclusion membrane by STIM1 recruitment supports extrusion formation (16). Interestingly, STIM1 was not detected at *C. psittaci* inclusions by indirect immunofluorescence microscopy (data not shown), and *C. psittaci* extrusion formation was not observed. This suggests that the absence of STIM1 at the *C. psittaci* inclusion membrane may facilitate *C. psittaci* inclusion membrane lysis, thus promoting CCS formation.

In recent years, several studies have demonstrated that the rupture of the *C. trachomatis* inclusion membrane is associated with the cytotoxicity of the infected host cell. However, the role of distinct cell death pathways during chlamydial infections is still under investigation and has been the subject of controversy (26, 27, 48–50). Interestingly, CCS formation also results in a form of host cell death, which shares some features of apoptosis, but lacks other features of apoptosis. The role of apoptosis in CCS formation was supported by the observation that the intracellular calcium concentration increases during CCS formation, plasma membrane blebbing, and exposure of phosphatidylserine at the CCS membrane surface. However, the following observations are not typical for apoptosis: active caspase-3 is absent, CCS formation could not be inhibited by different caspase inhibitors, including the pan-caspase inhibitor z-VAD-fmk, and nuclear condensation and fragmentation were not detected in CCSs.

Interestingly, the detachment of the infected host cell during CCS formation also occurs without damaging the surrounding cell monolayer and delays the release of host cell debris. Thus, we speculate that CCS formation could reduce the activation of the host defense system and tissue inflammation during *C. psittaci* infections. Additionally, as previously described and discussed in the context of *C. trachomatis* extrusions, the appearance of PS at the outer leaflet of the CCS membrane could potentially result in CCS being mistaken for apoptotic cells and thus being taken up by dendritic cells or other macrophages in *C. psittaci*-infected tissues (47, 54). This could subsequently influence the function of those immune cells. Taken together, this suggests a link between CCS formation and pathogenicity of *C. psittaci* and should therefore be studied in the future.

Taken together, we demonstrate that *C. psittaci* predominantly egresses by the novel non-lytic mechanism of CCS formation. The process of caspase-independent cleavage of a DEVD-containing substrate, calcium influx, inclusion membrane lysis, plasma membrane blebbing, and detachment of the whole infected host cell represents a new egress strategy for intracellular pathogens. In particular, this novel non-lytic egress mechanism is distinct from extrusion formation, the previously described non-lytic egress mechanism of the related but strict human pathogen *C. trachomatis*. Thus, we speculate that CCS formation could be linked to the specific biology of the zoonotic pathogen *C. psittaci*. Future studies in more complex cell culture models are needed to shed light on the benefits of specific egress strategies exploited by distinct chlamydial pathogens.

## SUPPLEMENTAL MATERIAL

Video S1 mbio.01288-24-s0001.mp4 Representative movie of CCS formation during *C. psittaci* infection.

Video S2 mbio.01288-24-s0002.mp4 Representative movie of extrusion formation during *C. trachomatis* L2 infection.

Video S3 mbio.01288-24-s0003.mp4 Representative movie of host cell lysis during *C. psittaci* infection.

Video S4 mbio.01288-24-s0004.mp4 Representative movie of CCS formation during *C. trachomatis* L2 infection.

Video S5 mbio.01288-24-s0005.mp4 Representative movie of host cell lysis during *C. trachomatis* L2 infection.

Supplemental material mbio.01288-24-s0006.pdf Figures S1 to S9, captions for Videos S1 to S5, and Text S1.

ASM does not own the copyrights to Supplemental Material that may be linked to, or accessed through, an article. The authors have granted ASM a non-exclusive, world-wide license to publish the Supplemental Material files. Please contact the corresponding author directly for reuse.
