# Supplementary material for: Chlamydia-containing spheres are a novel and predominant form of egress by the pathogen Chlamydia psittaci
Source: mBio. 2024 Jul 23;15(8):e01288-24. doi: 10.1128/mbio.01288-24 (PMC11323529; doi:10.1128/mbio.01288-24)

Appendix

Supplementary Figures

Supplementary Figure S1. Representative fluorescent image of a *C. trachomatis* L2 extrusion. HeLa cells were infected with GFP-expressing *C. trachomatis* L2 (MOI 2) and at 70 h pi, cells were stained with Hoechst. At 74 h pi, supernatants were microscopically analyzed; n = 3.

Supplementary Figure S2. Transmission electron microscopy of *C. psittaci*-infected cells and cellular structures found in the supernatant. Representative transmission electron microscopy images of thin sections through chemically fixed *C. psittaci*-infected HeLa cells (MOI 2, 48 h pi) **(A-C)** and *C. psittaci* containing cellular structures found in the supernatant above these cells **(D-F)**. **(A, B)** Section profiles of intact HeLa cells with intact inclusion bodies. The cell in (A) was fixed while adhered at the substrate (note the flat cell bottom) and the cell with the round profile in (B) was fixed in the supernatant. **(C)** Round cell profile of an apoptotic cell with a bacterial inclusion fixed in the supernatant. Note the profiles of the fragmented nucleus (arrow) which are surrounded by cisternae of the rough endoplasmic reticulum and the many profiles of larger vesicles. **(D-E)** Section profile of CCSs fixed in the supernatant with either visible (D) or invisible (E) plasma membrane. **(F)** Profiles of bacteria and cell organelles dispersed in low-melting point agarose used for stabilizing the sediment after fixation of the supernatant.

Supplementary Figure S3. Chemical inhibition of *C. trachomatis* L2 extrusion formation; *C. psittaci* CCS formation in A549 cells. (A) *C. trachomatis* L2 extrusion formation is influenced by inhibitors of host cell cytoskeleton elements. HeLa cells were infected with GFP-expressing *C. trachomatis* L2 at MOI 2, washed with PBS at 70 h pi, treated with 1 µM jasplakinolide, 0.5 µM latrunculin B, 30 µM nocodazole, 50 µM blebbistatin or DMSO (negative control) and stained with Hoechst. *C. trachomatis* L2 extrusions in the supernatant were quantified at 74 h pi. Data show mean ± SEM; n ≥ 3; $^*p < 0.05$; (Student's t-test). (**B)** CCS are formed in *C. psittaci*-infected A549 cells. Representative fluorescence images of *C. psittaci*-infected A549 cells (MOI 2, 24 h p.i). PFA-fixed cells were stained for *C. psittaci* and the inclusion membrane using a mouse-anti-Hsp60 (Cy3) and a rabbit-anti-IncA (AF488) antibody, respectively. DNA was counterstained using DAPI. n = 2. **(C)** Representative image of a CCS in the supernatant of infected A549 cells (MOI 2, 48 h pi); n = 3.

Supplementary Figure S4. Distribution of egress pathways of *C. trachomatis* L2. *C. trachomatis* L2 egress events of all egressing *C. trachomatis* L2-infected cells between 42 and 74.5 h pi (see supplemental Figure 1 A) **(A)** or within the last 4 hours of observation between 70.5 and 74.5 h pi **(B)** were quantified; n = 2.

Supplementary Figure S5. In contrast to apoptotic cells, CCS retain their membrane integrity. (A) Representative images of a CCS negative for SYTOX Green in comparison to staurosporine-treated,

apoptotic cells positive for SYTOX Green. CCS were collected from the supernatant of *C. psittaci*-infected HeLa cells (MOI 2, 48 h pi), while apoptotic cells were collected from the supernatant of uninfected, staurosporine-treated HeLa cells (10 µM staurosporine, 42 h post treatment). Structures with damaged or compromised membranes were stained with SYTOX Green and DNA was counterstained using Hoechst; n = 3. **(B)** Quantification of membrane integrity of CCS and staurosporine-treated, apoptotic cells. Mean intensities of the SYTOX Green staining in Hoechst-positive nuclei of CCS and of Hoechst-positive structures in apoptotic cells were determined and the distribution of the mean intensities of 54 CCS and 5515 apoptotic cell structures was visualized as violin plot; n = 3; ***p < 0.005 (Student's t-test).

Supplementary Figure S6. Transmission electron microscopy of a CCS and a staurosporine-treated, apoptotic cell. Representative transmission electron microscopy images of thin sections through a CCS isolated from the supernatant of *C. psittaci*-infected HeLa cells (MOI 2, 48 h pi) **(A, C, D)** and a staurosporine-treated, apoptotic cell **(B, E, F)**. **(A)** The CCS shows many bacterial profiles at different stages (RBs, EBs, intermediate forms) and cellular organelles, such as nucleus (n), mitochondria (m) and endoplasmic reticulum (er), that appear extracted and dilated (see **C** and **D** for a higher magnification). **(B)** The apoptotic cell reveals a dense cytoplasm with one larger and few smaller profiles of the fragmented nucleus (asterisk). The matrix of the nuclear fragments appears dense and homogenous. Nuclear membranes (arrowheads), with typical nuclear pore structures, are detached and located in the adjacent cytoplasm **(E)**. The mitochondria reveal an aberrant internal structure, with condensed matter and/or vesicular structures **(F)**.

Supplementary Figure S7. Z-VAD-FMK and Z-DEVD-FMK inhibit caspase 3 during staurosporine-induced apoptosis of HeLa cells in concentrations between 2 and 50 µM. HeLa cells were washed with PBS, treated with Z-VAD-FMK and Z-DEVD-FMK in concentrations of 2, 20 and 50 µM or left untreated. At 4 h post treatment, apoptosis was induced by addition of 5 µM staurosporine and caspase 3 activity was monitored using Incucyte Caspase-3/7 Red Dye for Apoptosis. Representative fluorescence images of each condition are shown; n = 2.

Supplementary Figure S8. Proteolytic cleavage of DEVD can be observed in *C. psittaci*-infected cells after treatment with Z-DEVD-FMK. *C. psittaci*-infected HeLa cells (MOI 2, 40 h pi) were treated with 2 µM Z-DEVD-FMK to inhibit caspase-3 or left untreated (DMSO control). Between 44 and 48 h pi, proteolytic cleavage of DEVD was monitored using Incucyte Caspase-3/7 Red Dye for Apoptosis. Representative fluorescence images of both conditions at 48 h pi are shown; n = 2.

Supplementary Figure S9. Intracellular calcium concentration increases during CCS formation (A, B) HeLa cells were infected with *C. psittaci* (MOI 2). At 44 h pi, medium was replaced by serum-free medium supplemented with 0 mM calcium chloride **(A)** or 1.8 mM calcium chloride **(B)**. At 46 h pi, cells

were labeled with the calcium sensor Rhod-3 for 1 h. At 48 h pi, DNA was counterstained using Hoechst and images were acquired. Representative images of intact cells (0.0 mM $CaCl_2$) and CCS formation (1.8 mM $CaCl_2$) are shown; n = 2.

Supplementary Movies

Supplementary Movie S1. Representative movie of CCS formation during *C. psittaci* infection. *C. psittaci*-infected HeLa cells (MOI 2) stably expressing GFP were monitored using a CLSM equipped with a live-cell chamber. n = 3.

Supplementary Movie S2. Representative movie of extrusion formation during *C. trachomatis* L2 infection. *C. trachomatis* L2-infected HeLa cells (MOI 2) stably expressing GFP were monitored using a CLSM equipped with a live-cell chamber. n = 3.

Supplementary Movie S3. Representative movie of host cell lysis during *C. psittaci* infection. *C. psittaci*-infected HeLa cells (MOI 2) stably expressing GFP were monitored using a CLSM equipped with a live-cell chamber. n = 3.

Supplementary Movie S4. Representative movie of CCS formation during *C. trachomatis* L2 infection. *C. trachomatis* L2-infected HeLa cells (MOI 2) stably expressing GFP were monitored using a CLSM equipped with a live-cell chamber. n = 3.

Supplementary Movie S5. Representative movie of host cell lysis during *C. trachomatis* L2 infection. *C. trachomatis* L2-infected HeLa cells (MOI 2) stably expressing GFP were monitored using a CLSM equipped with a live-cell chamber. n = 3.

Supplementary Experimental Procedures

S1 Text

Cell culture, transient transfection and infection assays

Cell culture, transient transfection and infection assays were performed as described previously (Koch-Edelmann et al., 2017). Briefly, HeLa cells (ATCC CCL-2) and A549 cells (ATCC CCL-185) were cultivated in RPMI 1640 medium (Gibco) and Ham's F-12K (Kaighn's) medium (Gibco), respectively, supplemented with 10% fetal bovine serum (FBS, Sigma-Aldrich), 1 mM sodium pyruvate (Gibco), and 2 mM L-glutamine (Gibco) at 37°C and 5% CO_2 and passaged every 2 to 3 days. Transient transfection was performed 4 h prior to infection with Lipofectamine 2000 reagent (Thermo Fisher) according to manufacturer's instructions. For infection assays, subconfluent HeLa cells and A549 cells were washed with infection medium (Dulbecco's modified Eagle's medium (DMEM, Gibco) with glucose (4.5 g L^{-1}) supplemented with 5% FBS, 1 mM sodium pyruvate, and 2 mM L-glutamine) and A549 infection medium, (Ham's F-12K (Kaighn's) medium supplemented with 5% FBS, 1 mM sodium pyruvate, and 2

mM L-glutamine) respectively, and incubated at 35°C and 5% CO_2 using the indicated multiplicity of infection (MOI) with *C. psittaci* strain 02DC15 (Goellner et al., 2006), *C. trachomatis* L2 lymphatic isolate 434 Bu (ATCC VR-902B), or GFP-expressing *C. trachomatis* L2/434/Bu strain transformed with pGFP::SW2 kindly provided by Thomas Rudel. *C. psittaci*-infected cell cultures were centrifuged (30 min, 600 x *g*, room temperature (RT)) at 30 min pi. *C. psittaci* and *C. trachomatis* L2-infected cell cultures were washed with infection medium at 2 h pi.

Live cell imaging of *C. psittaci* and *C. trachomatis* egress

To monitor *C. psittaci* or *C. trachomatis* egress, live cell imaging of *Chlamydia*-infected cells stably expressing GFP was performed.

HeLa cells stably expressing eGFP were cultured and infected in 8 well chambered coverslips (µ-Slide 8 Well, Ibidi). Live cell microscopy was performed at a Stellaris 8 Confocal Microscope (Leica Microsystems) equipped with a live cell chamber at 35°C. Z-stacks of 12 slices with each 3 µm distance covering both adherent cells and the supernatant containing CCS and extrusions were acquired every 2:10 minutes for a total of 32.5 hours starting 42 h pi. For live cell imaging of *C. psittaci* egress under calcium-free conditions, cell cultures were washed with PBS at 42 h pi and calcium- and FBS-free infection medium was added for live imaging. Live cell imaging data was visually analyzed for the type of egress pathway (CCS formation, lysis, extrusion formation or no egress, see movies S1-S5 for an overview of the egress pathways) and time point. In total, for *C. psittaci* egress, *C. trachomatis* egress, and *C. psittaci* egress under calcium-free conditions, 410, 391, and 427 infected cells were analyzed, respectively.

Determination of the working concentrations of the caspase inhibitors

To determine the working concentration of the caspase-3 inhibitor Z-DEVD-FMK and the pan caspase inhibitor Z-VAD-FMK in our cell culture model, HeLa cells were seeded in 8 well chambered coverslips (µ-Slide 8 Well, Ibidi). Cells were treated with inhibitors diluted into RPMI in concentrations of 0, 2, 20, and 50 µM. At 4 h post treatment, apoptosis was induced by addition of 5 µM staurosporine (Biomol) and caspase-3 activity was monitored using Incucyte Caspase-3/7 Red Dye for Apoptosis (Sartorius) in a final assay concentration of 833 nM. At 16 h post staurosporine treatment, samples were analyzed using an LSM 780 CLSM (Carl Zeiss).

Isolation and staining of CCS

CCS were analyzed using different dyes. FM4-64 and Hoechst 33342 were used to stain the membrane and nucleic acids, respectively. SYTOX Green and Trypan Red Plus are both impermeable to intact cellular membranes but can permeate the compromised membranes of dead cells and were used to study the membrane integrity of CCS. While SYTOX Green is a nucleic acid dye, Trypan Red Plus stains

the whole damaged cell. AnnexinV is a protein binding PS with high affinity and is used to detect the exposure of PS to the outer leaflet of CCS membrane. CCS in the supernatant of *C. psittaci*-infected cell cultures were separated by centrifugation (5 min, 300 x *g*, RT). For live staining, pellet was mixed with the indicated staining solutions. The following staining solutions were used: 10 mM FM4-64 in DMSO (Sigma-Aldrich) diluted 1:1000 in infection medium; 25 mg mL^{-1} Hoechst 33342 (Sigma-Aldrich) diluted 1:2000 in infection medium; 5 mM SYTOX Green in DMSO (Thermo Fisher Scientific) diluted 1:2000 in infection medium; annexin V Alexa Fluor 568 (Thermo Fisher Scientific) diluted 1:10 in annexin binding buffer (PBS supplemented with 400 mg L^{-1} $CaCl_2$); 100 mM Trypan Red Plus (AAT Bioquest) diluted 1:100 in PBS. CCS were transferred into a 15 well chambered coverslip (µ-Slide 15 Well 3D, Ibidi) and either examined directly (FM4-64, Hoechst, SYTOX and Trypan Red Plus staining) or examined after 15 min of incubation at RT (annexin V staining) using an LSM 780 CLSM (Carl Zeiss). For immunofluorescence staining, pellet was mixed with 4% paraformaldehyde in PBS and transferred into a poly-L-lysine coated 8 well chambered coverslip (µ-Slide 8 Well, Ibidi). After 30 min of incubation at RT, staining was continued as described for immunofluorescence assays.

Antibodies used for immunofluorescence assay

Antibodies were used at the following concentrations: mouse anti-Hsp60 (chlamydial, cHsp60; 1:600, Cat. No. ALX-804-072, Enzo Life Sciences), rabbit anti-HaloTag (1:500, Cat. No. G9281, Promega), rabbit anti-cleaved caspase-3 (1:400, Cat. No. 9661, Cell Signaling Technology), rabbit anti-IncA (1:500), Alexa Fluor 488-coupled goat anti-rabbit IgG (1:100, Cat. No. 111-545-144, Dianova), and Cy3-coupled goat anti-mouse IgG (1:200, Cat. No. 115-165-146, Dianova). Polyclonal rabbit anti-IncA antibody were produced by immunization of rabbits with the C-terminal cytoplasmic fragment of IncA fused to GST. All animal handling and antibody purification was performed by Biogenes, Berlin. Antigens were produced in *Escherichia coli* Rosetta 2 (Merck) transformed with the Inc-GST fusion protein expression vectors described above. Expression of GST-IncA fusion protein was induced by addition of Isopropyl β-D-1-thiogalactopyranoside (IPTG, Roth). After protein expression, bacteria were pelleted and the pellets were incubated with lysis buffer (50 mM Tris, 0.1% Triton X100, 100 µg/mL lysozyme, 500 U/µL nuclease, pH 8.0, with Complete protease inhibitor cocktail (Sigma Aldrich)) for 30 min on ice, followed by 5x 30 s sonification interrupted by incubation on ice for 30 s to lyse bacteria and thereby release the GST-IncA fusion protein. After centrifugation (12.000 x g, 20 min, 4 °C), the supernatant containing the soluble fraction of the GST-IncA fusion protein was added to a 5 mL disposable plastic column (Thermo Scientific) filled with prewashed Glutathion-Sepharose 4B (Sigma-Aldrich) and incubated at 4 °C under rotation to bind the GST-IncA fusion protein to the glutathione sepharose beads. The beads were washed three times with PBS and the Inc-GST fusion protein was eluted from the beads by incubation with elution buffer (50 mM Tris-HCl, 10 mM reduced glutathione, pH 8.0) at RT for 20 min three times.

Imaging cytosolic sphingomyelin (SM) exposure

To monitor the cytosolic SM exposure at the inclusion membrane, we used a HeLa cell line stably expressing a non-toxic, Halo-tagged variant of equinatoxin II (EqtSM-HaloTag) (Niekamp et al., 2022). Cytosolic exposure of SM was determined by immunofluorescence assays at middle and late infection time points. For this, cells were fixed at 24 and 48 h pi and stained as described for immunofluorescence assays. To determine cytosolic exposure of SM under caspase inhibition, the caspase inhibitors Z-VAD-FMK (R&D-Systems) and Z-DEVD-FMK (R&D-Systems) were both used in concentrations of 2 µM. In addition, experiments with calcium- and FBS-free infection medium supplemented with the 0 or 1.8 mM of calcium chloride (Roth) were performed. At 44 h pi, cell cultures were washed with PBS and medium was changed to the described medium conditions. Cultivation was continued for additional 4 h and subsequently, cells were fixed and stained as described for immunofluorescence assays. In addition, live cell imaging experiments were performed. For this, cell culture, transient transfection and infection were performed in 8 well chambered coverslips (µ-Slide 8 Well, Ibidi). HeLa cells stably expressing EqtSM-HaloTag were transiently transfected with a plasmid for cytosolic expression of eGFP (pEGFP-N1, Clonetech) and infected with *C. psittaci* (MOI 2). At 43.5 h pi, cells were labeled with 200 nM Janelia Fluor 585 HaloTag Ligand (Promega) in infection medium for 30 min, washed with infection medium and live cell imaging was performed at a Stellaris 8 Confocal Microscope (Leica Microsystems) equipped with a live cell chamber at 35°C. Z-stacks of 12 slices with each 3 µm distance were acquired every 2.5 minutes for a total of 4 hours.

Quantification of CCS or extrusions

The number of CCS or extrusions in *C. psittaci*- or *C. trachomatis*-infected cell cultures, respectively, was determined under different inhibitory and medium conditions. The following inhibitors were used: blebbistatin (Sigma-Aldrich, 50 µM), jasplakinolide (Santa Cruz, 1 µM), latrunculin B (Merck, 0.5 µM), nocodazole (Sigma-Aldrich, 30 µM), wiskostatin (Sigma-Aldrich, 50 µM), Z-VAD-FMK (R&D-Systems, 2 µM), Z-DEVD-FMK (R&D-Systems, 2 µM), and BAPTA-AM (Merck, 10 and 20 µM). In addition, experiments with calcium- and FBS-free infection medium (Cat. No. 21068028, Gibco) supplemented with the indicated concentrations of calcium chloride (Roth) were performed. At indicated time points, cell cultures were washed with PBS and medium was changed to conditional medium and cultivation was continued for additional 4 h. To quantify the number of CCS or extrusions, supernatant of the infected cell cultures was transferred to a new 12 well plate. The average number of CCS or extrusions of at least five visual fields was determined and normalized to the infected cell area.

Determination of proteolytic DEVD cleaving activity

Proteolytic DEVD cleaving activity was determined using the Incucyte Caspase-3/7 Red Dye for Apoptosis (Sartorius). Inert Incucyte Caspase-3/7 Red Dye for Apoptosis is a membrane permeable,

non-fluorescent reagent consisting of the activated caspase-3/7 recognition motif DEVD coupled to a DNA intercalating dye. Cleavage of the DEVD motif by caspase-3/7 or other DEVD cleaving proteases releases the DNA intercalating dye and results in red DNA staining. To determine proteolytic DEVD cleaving activity, cells were cultured and infected in 8 well chambered coverslips (µ-Slide 8 Well, Ibidi). Proteolytic DEVD cleaving activity was determined using the Incucyte Caspase-3/7 Red Dye for Apoptosis (Sartorius) as described in the manufacturer's protocol. Briefly, Incucyte Caspase-3/7 Red Dye was diluted in infection medium to a final assay concentration of 833 nM and added to *C. psittaci*-infected cell cultures at indicated time points. After further cultivation (35°C, 5% CO_2), samples were analyzed using an LSM 780 CLSM (Carl Zeiss).

Calcium imaging

Imaging of intracellular calcium levels and distribution was performed by either Rhod 3 staining or live cell imaging of the dual calcium reporter cell line HeLa ER-LAR-Geco G-Geco (Stelzner et al., 2020).

The red-fluorescent Rhod-3 AM dye is a plasma membrane permeable dye that shows hardly no uptake to cellular organelles and thus localizes in the cytosol. After calcium-binding, the red fluorescence of Rhod-3 strongly increases. For Rhod 3 staining, cell culture and infection were performed in 8 well chambered coverslips (µ-Slide 8 Well, Ibidi). Rhod-3 staining was performed using the Rhod-3 Calcium Imaging Kit (Thermo Fisher Scientific) as described in the manufacturer's protocol. In brief, cells were washed with PBS twice and 150 µL per well of freshly prepared Rhod-3 loading buffer (1x PowerLoad concentrate, 10 µM Rhod-3 AM, 250 µM Probenecid in infection medium or calcium- and FBS-free infection medium) were added. After incubation (60 min, 35°C, 5% CO_2), cells were washed three times with PBS and 150 µL per well of incubation buffer (250 µM Probenecid in infection medium or calcium- and FBS-free infection medium) were added. After incubation (60 min, 35°C, 5% CO_2), cells were washed with PBS and 150 µL per well of 25 mg mL^{-1} Hoechst 33342 (Sigma-Aldrich) diluted 1:2000 in infection medium were added for live cell microscopy. Live cell microscopy was performed at a Stellaris 8 Confocal Microscope (Leica Microsystems).

For live cell calcium imaging, the dual calcium reporter cell line HeLa ER-LAR-Geco G-Geco (Stelzner et al., 2020) was cultured and infected in 8 well chambered coverslips (µ-Slide 8 Well, Ibidi). This cell line stably expresses a red fluorescent endoplasmic reticulum (ER)-targeted fluorescent calcium sensor (ER-LAR-Geco) in addition to a green fluorescent cytosolic calcium indicator (G-Geco). For imaging of cytosolic calcium levels, the green channel was monitored using a Stellaris 8 Confocal Microscope (Leica Microsystems) equipped with a live cell chamber at 35°C. Z-stacks of 13 slices with each 2.5 µm distance covering both adherent cells and CCS were acquired every 2.5 minutes for a total of 4 hours. For imaging of cytosolic calcium levels in combination with determination of the proteolytic DEVD cleaving activity, cells were stained with Incucyte Caspase-3/7 Red Dye for Apoptosis (Sartorius) as

described. Live imaging was performed using an LSM 780 CLSM (Carl Zeiss) equipped with a live cell chamber at 35°C. Z-stacks of 12 slices with each 2.5 µm distance covering both adherent cells and CCS were acquired every 2.5 minutes for a total of 4 hours.

Supplementary Figure S1

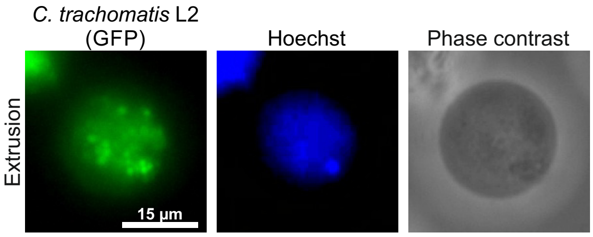

Supplementary Figure S2

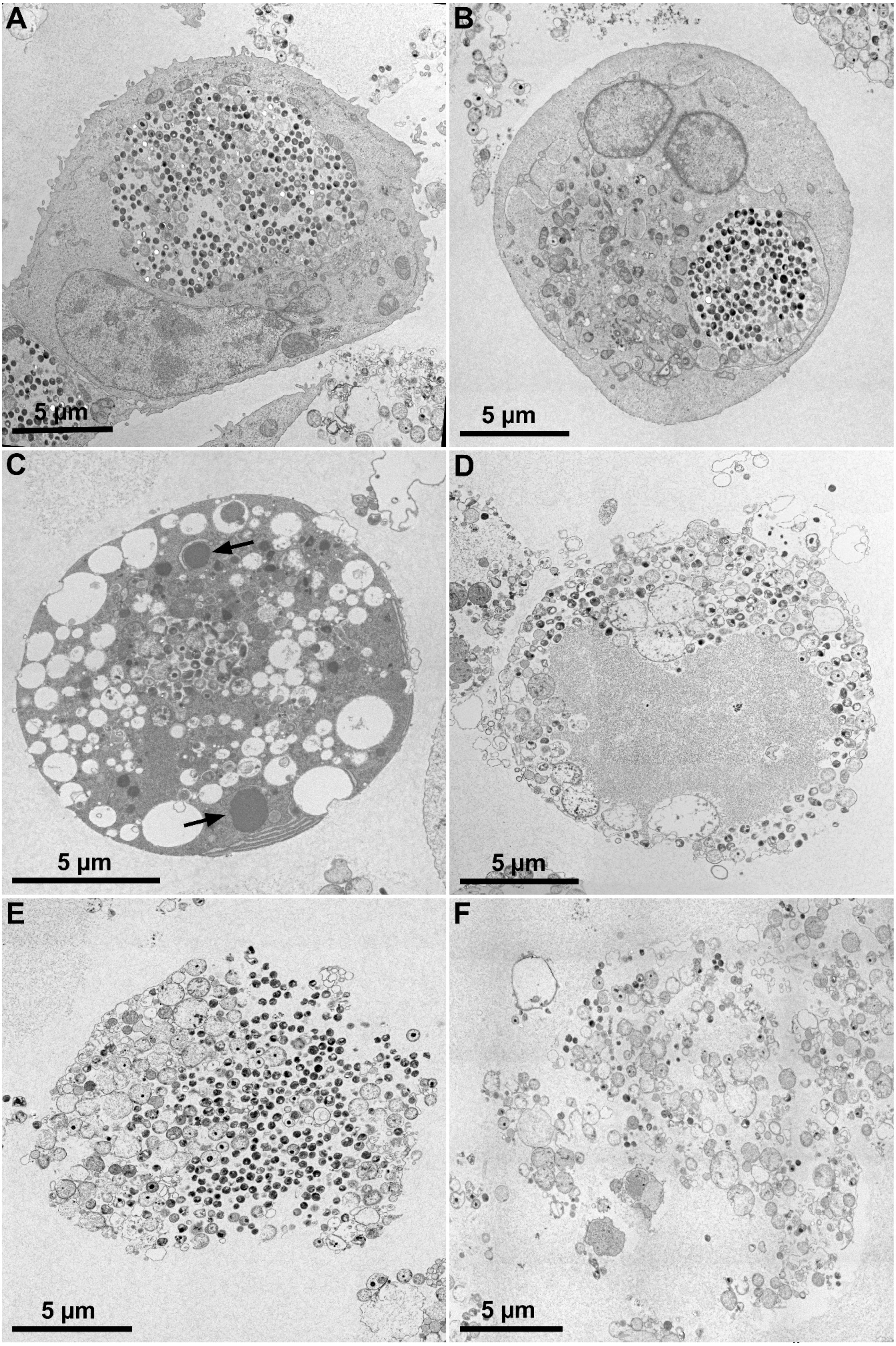

Supplementary Figure S3

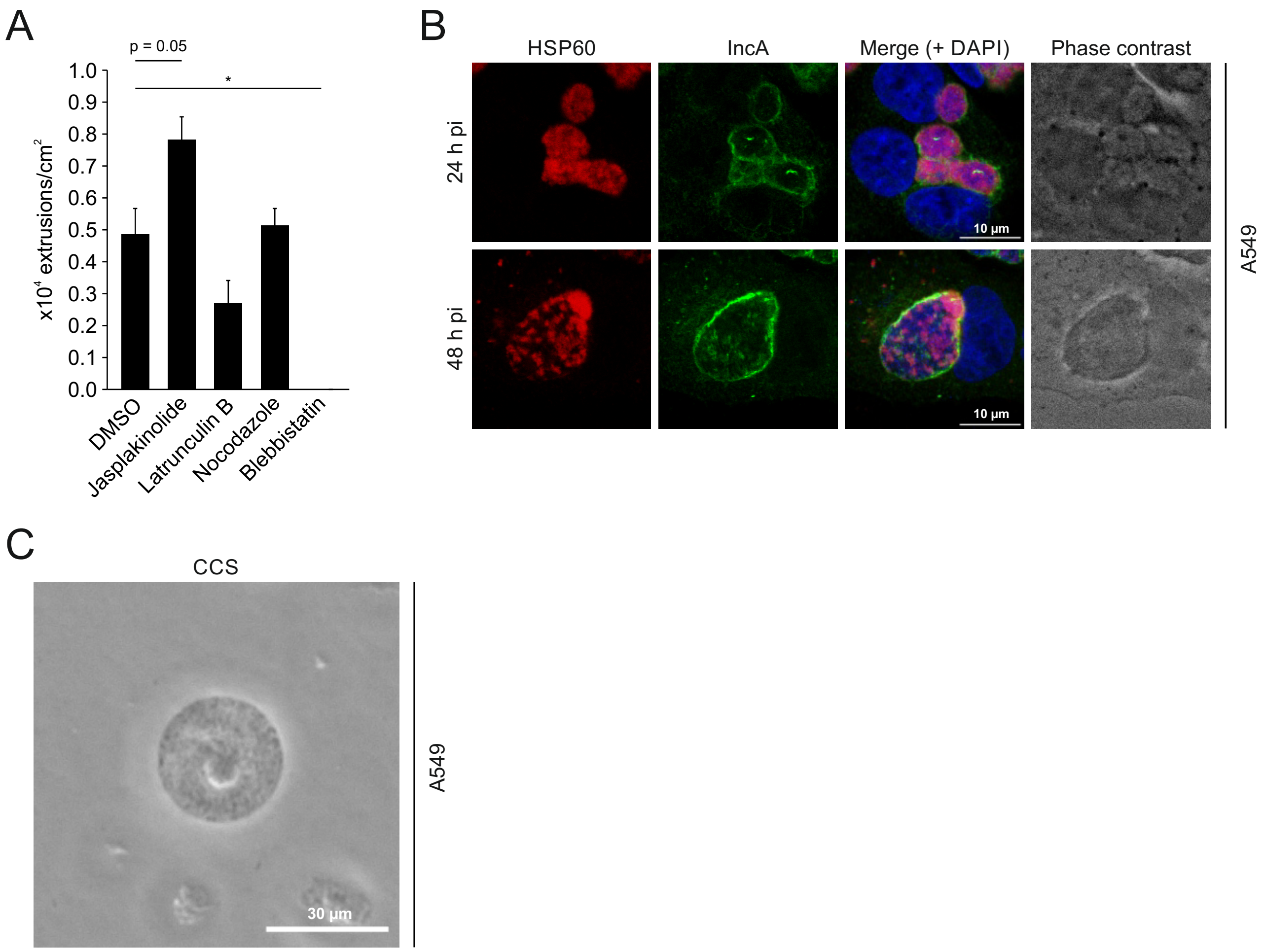

Supplementary Figure S4

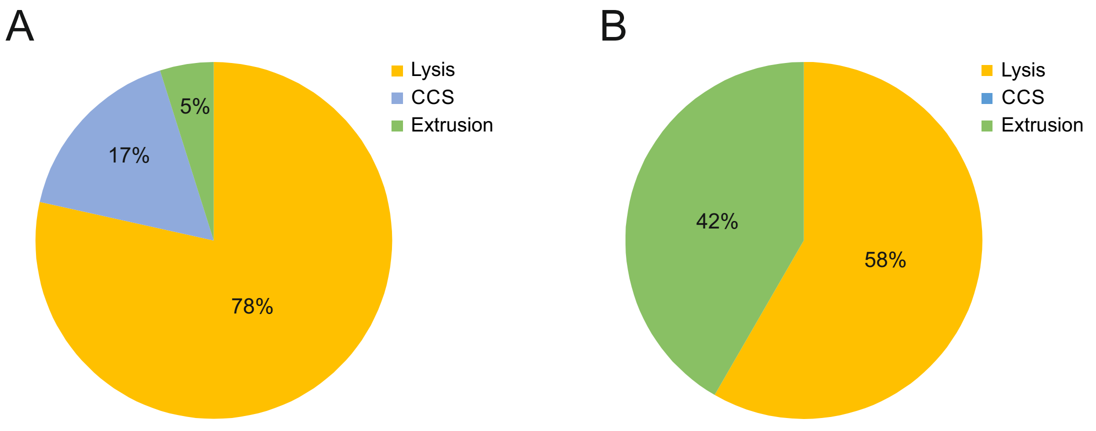

C. trachomatis L2
(42-74.5 h pi)

C. trachomatis L2
(70.5-74.5 h pi)

Supplementary Figure S5

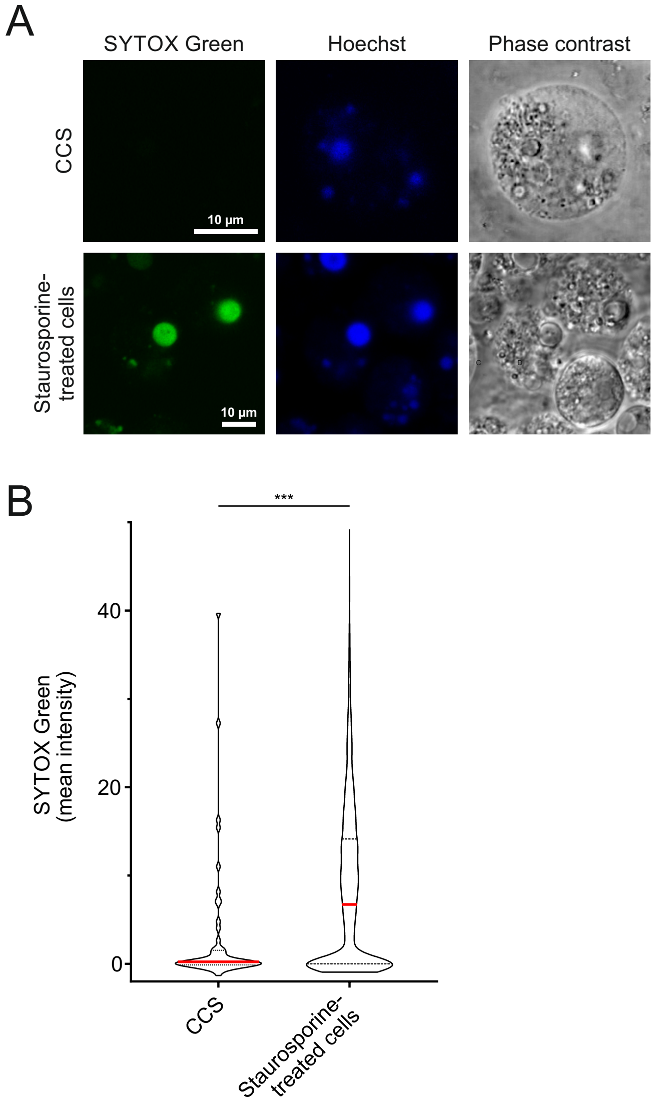

Supplementary Figure S6

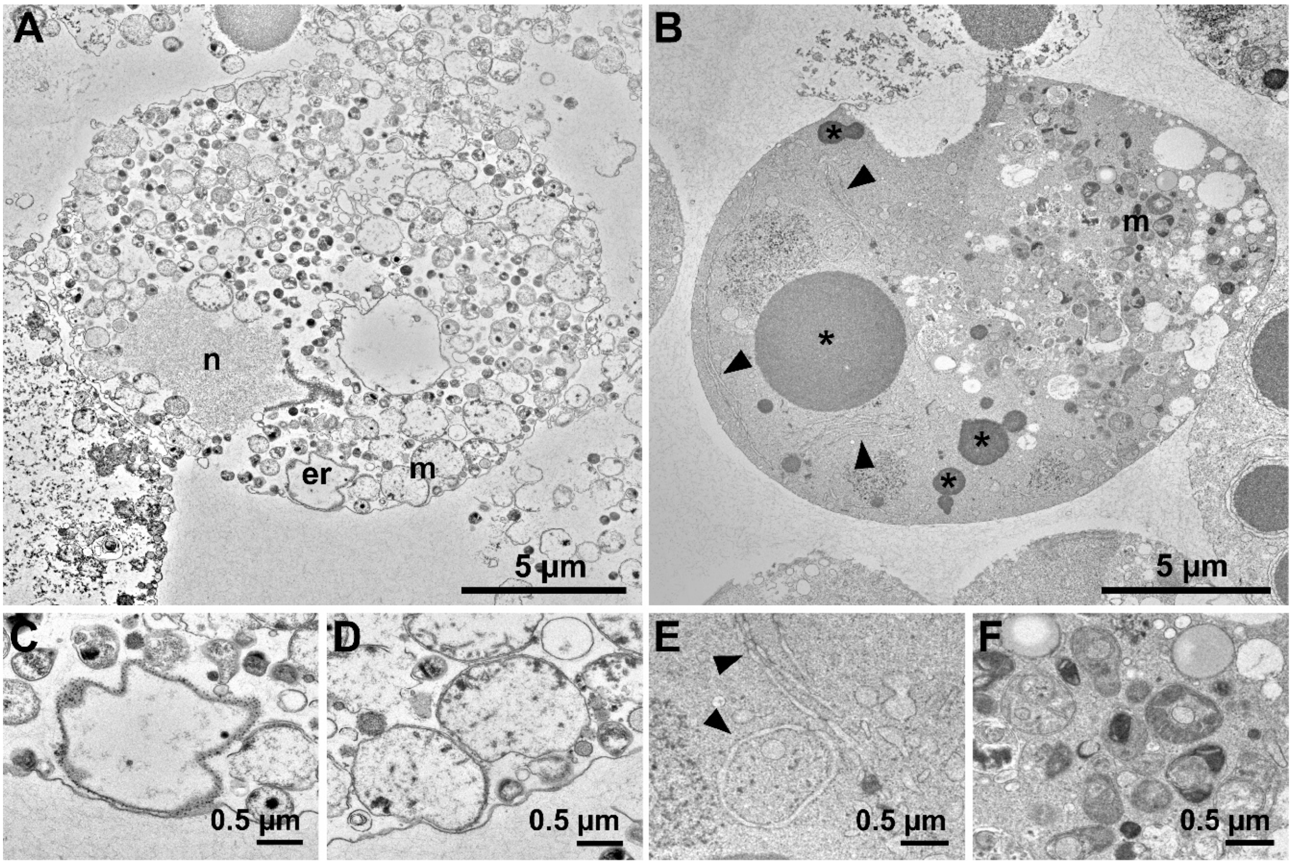

Supplementary Figure S7

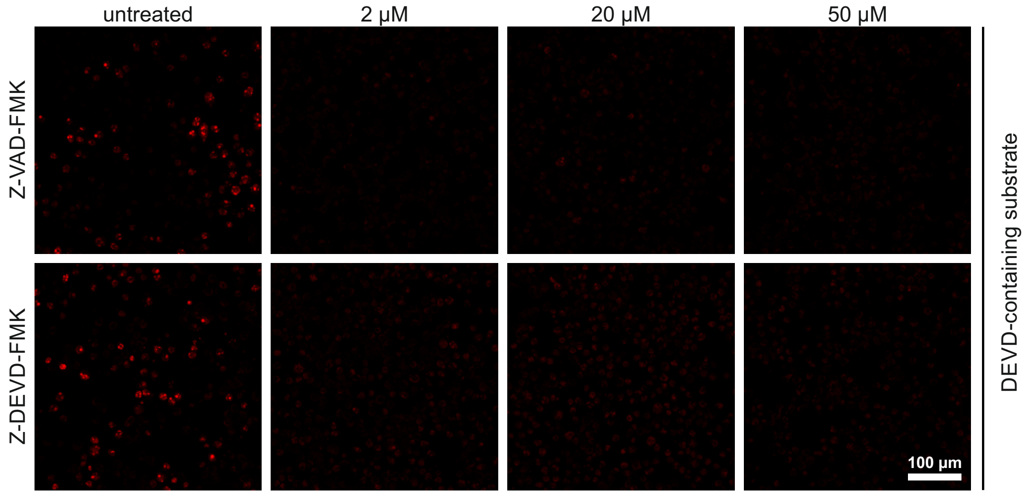

Supplementary Figure S8

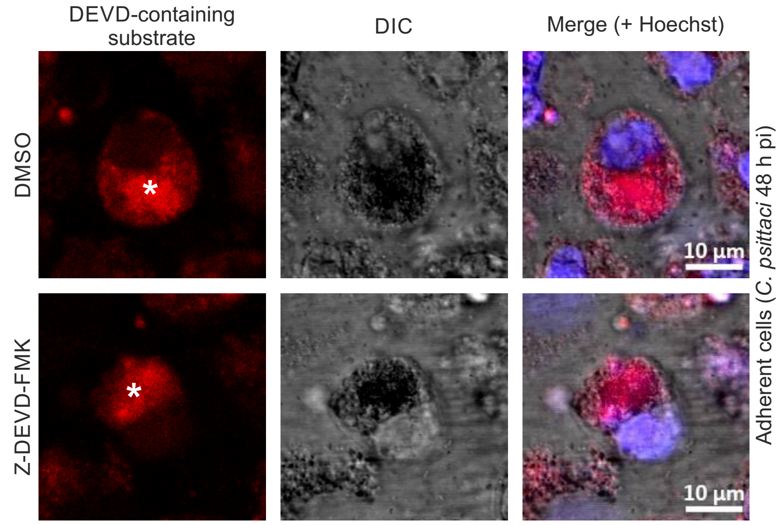

Supplementary Figure S9

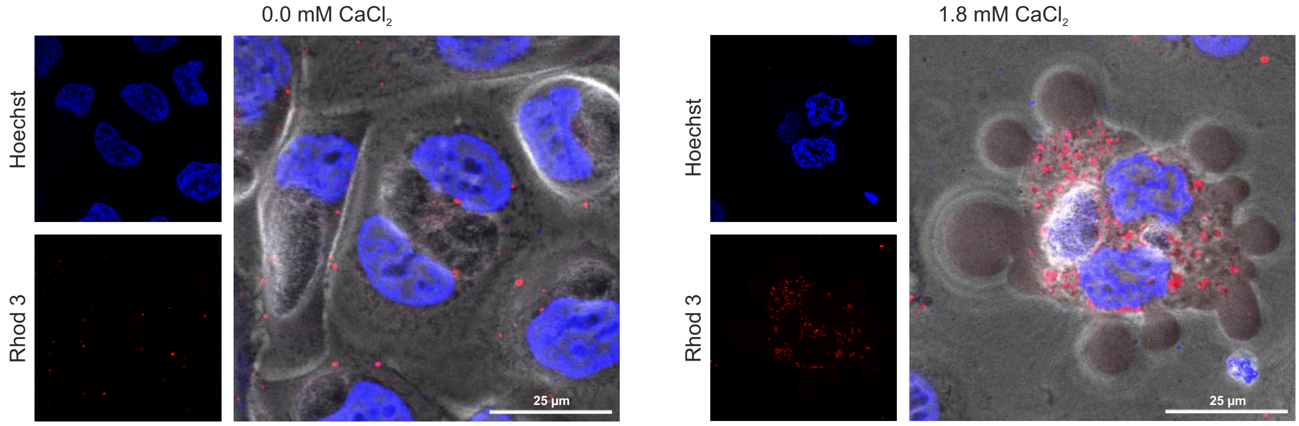

Supplement: Supplemental material — Figures S1 to S9, captions for Videos S1 to S5, and Text S1. [file mbio.01288-24-s0006.pdf]
